# How public can public goods be? Environmental context shapes the evolutionary ecology of partially private goods

**DOI:** 10.1371/journal.pcbi.1010666

**Published:** 2022-11-01

**Authors:** Brian A. Lerch, Derek A. Smith, Thomas Koffel, Sarah C. Bagby, Karen C. Abbott

**Affiliations:** 1 Department of Biology, University of North Carolina at Chapel Hill, Chapel Hill, North Carolina, United States of America; 2 Department of Biology, Case Western Reserve University, Cleveland, Ohio, United States of America; 3 W. K. Kellogg Biological Station, Michigan State University, Hickory Corners, Michigan, United States of America; Emory University Department of Biology, UNITED STATES

## Abstract

The production of costly public goods (as distinct from metabolic byproducts) has largely been understood through the realization that spatial structure can minimize losses to non-producing “cheaters” by allowing for the positive assortment of producers. In well-mixed systems, where positive assortment is not possible, the stable production of public goods has been proposed to depend on lineages that become indispensable as the sole producers of those goods while their neighbors lose production capacity through genome streamlining (the Black Queen Hypothesis). Here, we develop consumer-resource models motivated by nitrogen-fixing, siderophore-producing bacteria that consider the role of colimitation in shaping eco-evolutionary dynamics. Our models demonstrate that in well-mixed environments, single “public goods” can only be ecologically and evolutionarily stable if they are partially privatized (i.e., if producers reserve a portion of the product pool for private use). Colimitation introduces the possibility of subsidy: strains producing a fully public good can exclude non-producing strains so long as the producing strain derives sufficient benefit from the production of a second partially private good. We derive a lower bound for the degree of privatization necessary for production to be advantageous, which depends on external resource concentrations. Highly privatized, low-investment goods, in environments where the good is limiting, are especially likely to be stably produced. Coexistence emerges more rarely in our mechanistic model of the external environment than in past phenomenological approaches. Broadly, we show that the viability of production depends critically on the environmental context (i.e., external resource concentrations), with production of shared resources favored in environments where a partially-privatized resource is scarce.

## Author summary

Many organisms produce “public goods”, substances that may directly benefit their competitors as well as themselves. Because goods production is costly, understanding the evolutionary stability of public goods production has been a subject of considerable interest: what keeps cheaters from taking over a population and driving producers to extinction? Here, we ask when partial privatization of public goods (that is, when producers retain some portion of the good for their own exclusive use) is sufficient to stabilize production even in the absence of spatial structure, and how this depends on environmental conditions. We derive lower bounds for the amount of privatization needed to stabilize production and find that these bounds depend critically on environmental conditions. We further investigate the case of two public goods, each needed for the acquisition of the other, and each a resource whose availability limits growth. We find that the ecological dynamics of such colimiting resources can interact, with privatization of one resource subsidizing more-public, or even fully public, production of the other. Finally, we offer the perspective that producers are not “losers” in a race of loss-of-function mutations, but rather can do no better than to produce the resource in a given set of conditions.

## Introduction

The public goods dilemma—arising when free-riders exploit a shared resource pool built up by individuals making costly contributions—is a central problem in the evolution of cooperation. It appears in microbes [1–3], animals [4, 5], and humans [6, 7], and has received extensive theoretical attention [8–11]. Many mechanisms of stable public goods production require a means of positive assortment between producers, typically through spatial or metapopulation structure [1, 12–15], that allows producers to share mainly with other producers [16, 17]. But population structures are unlikely to be the answer to the public goods dilemma everywhere, for example in environments like the surface ocean, where spatial structure is often poorly defined or transient, but nonetheless public goods are produced [18, 19].

Theory considering the production of public goods in well-mixed systems has suggested that such goods are likely not completely public: the ability for the producer to retain some of the good is essential [20–22]. Such privatization is a general solution to prevent the tragedy of the commons [23] and has been shown empirically to carry fitness benefits in producers [20, 24, 25]. Past theory on partially privatized public goods developed phenomenological models that do not explicitly track changes in the extracellular environment. Consequently, this work cannot be connected to consumer-resource theory, a well-developed framework in theoretical ecology that has demonstrated the important role of the environment (and organisms’ modification of the environment) in shaping ecological and evolutionary outcomes [26–28]. Results from consumer-resource theory suggest that the benefits of producing partially privatized public goods should depend strongly on the extracellular environment—a possibility that cannot be assessed in previous phenomenological approaches.

The production of partially privatized goods that are both essential and expensive to produce is expected to be lost through genome streamlining [29, 30] if the goods are supplied by other organisms (the Black Queen Hypothesis (BQH); [31]). In the context of the BQH, partially privatized goods are referred to as “leaky” and lie along a spectrum from public to private: a secreted enzyme in a vigorously shaken dense culture is purely public, the product of a reaction in the periplasm or cell wall represents a midpoint, and a molecule produced and used in an intracellular compartment inaccessible to other cells is purely private [32]. Producers of the leaky good (or “helpers”) can have a fitness advantage when the product is rare in the environment, so long as production remains a net benefit. The BQH argues that purely selfish loss-of-function (LOF) mutants or “beneficiaries” that rely on helpers’ continued production of public goods gain an advantage as producers become common and vice versa, such that negative frequency dependence facilitates coexistence [32].

Two distinct types of public goods characterized by very different types of sharing could be subject to the BQH. The first, which we refer to as “public services”, are goods that benefit all individuals equally and are not drawn down by use, like detoxification enzymes (e.g., catalases) or some antibiotic resistance molecules. The second, which we refer to as “public consumables”, are classical resources (e.g., siderophores; fixed nitrogen) that cells can produce, extract from the environment, and use to their own exclusive benefit. In contrast with public services, public consumables are drawn down by non-producers, preempting producers’ use. Public services’ potential to shape ecological interactions and microbial community assembly has been treated in a formal model [22]. The key driver of ecological outcomes in that model is privatization: as the service becomes more private, producing cell strains become better competitors and exclude LOF mutants.

As with other models of partial privatization, no mechanistic model for public consumables exists in the context of the BQH (but see [33] and [34] for related work in plants), despite the fact that the exclusive benefit provided by using public consumables may result in fundamentally different ecology and evolution compared to public services. Unlike public services, the dynamics of public consumables and the cells that use them relate to classic resource-explicit models (e.g., [26, 28, 35]), which have explained how external resource supplies and environmental feedbacks shape ecological and evolutionary outcomes. Importantly, this suggests an approach to the problem of facultative producers, which can cease production in favor of direct acquisition of an available public good [36–41]. Facultative producers blur the lines between producers and beneficiaries, thereby complicating the application of phenomenological explanations for negative frequency dependence in BQH systems [31, 32]. Consumer-resource theory also provides a well-developed means of accounting for a second key influence on microbial growth: colimitation, in which multiple elements simultaneously limit growth [42, 43]. Connecting public consumables to consumer-resource theory should thus permit construction of a model that extends beyond the single production pathways considered in [31] and the simplified multi-resource view (e.g. additive in effect, equivalent in cost and leakiness) employed by [22], toward a clearer understanding of the environmental contexts that allow Black Queen dynamics emerge in multi-resource systems of facultative producers. Doing so would provide a mechanistic understanding of the trade-offs involved in colimited systems, such as whether producing one highly privatized resource can compensate for producing a second, highly public resource.

Here, we aim to understand the importance of (1) the external environment and (2) interactions between multiple, colimiting resource pathways with different levels of privatization in shaping the evolutionary ecology of partially privatized public goods. We develop a pair of mechanistic consumer-resource models to analyze the role the environment plays in the evolutionary ecology of partially privatizated public goods production. We begin with a simple model that allows us to confirm that the conditions under which privatization can stabilize public goods production hold when the external environment is explicitly modeled. Then, motivated by a case study of public goods production in planktonic marine cyanobacteria, we build a more realistic (and more complex) model with two resources that colimit cell growth in a perfectly well-mixed system. Considering a well-mixed environment allows us to discover whether spatial structure is necessary, or whether partial privatization is itself a viable mechanism for stabilizing public goods production. We find that, in the absence of spatial structure, private benefits (incomplete leakiness of public goods) are needed to stabilize the production of costly, consumable public goods. We derive a lower bound on what proportion of the good must be privatized to yield a competitive benefit and find that this depends critically on the level of resource in the external environment. Notably, private benefits from one pathway’s production of a partially leaky consumable can even subsidize a second, linked pathway’s production of a fully public good.

## Private benefits to public goods production in resource-explicit models

We begin with a simple consumer-resource model involving the investment into producing a consumable public good that is partially privatized. We use this model to build intuition and acquire analytical conditions for how privatization influences the competition between producers and LOF mutants in different environments. In particular, the single resource model allows us to ensure that past theoretical conclusions about privatization [20, 21] hold while explicitly modeling the extracellular environment and treating the system as well mixed rather than diffusive (i.e., as a system in which any resource that is not kept within the cell is equally likely to be obtained by any cell in the population). The goal of the single-resource model is simplicity and generality; thus, it is abstract. In the following section, we build on insights from the single-resource model to develop a case study of marine cyanobacteria that includes considerably more biological detail.

### Single-resource model formulation

We model two cell populations with densities *x*_1_ and *x*_2_, respectively, limited by the same resource with extracellular concentration *R*. For simplicity, we model resource concentrations in units that correspond to cell growth (i.e., the stoichiometric coefficient is 1 such that obtaining 1 unit of resource increases per capita growth rate by 1). To connect this model to any real system would thus require a proper conversion factor of units of resource to units of cell growth; because a constant conversion factor would cause no qualitative changes to our results, we omit it for simplicity. The cell populations grow according to,

$$\begin{array}{c} \frac{dx_{i}}{dt}=(g_{i}\left(R\right)-\delta )x_{i} \end{array}$$ (1)

for strains *i* = 1, 2. The function *g*_*i*_(*R*) describes the per capita population growth of strain *i* in an environment with resource level *R*, based on that strain’s ability to produce and/or take up resource. Both strains die or are washed out of the system at per capita rate *δ* (parameters are summarized in Table 1).

**Table 1:** Summary of model parameters and functions for both models (SR = single resource model, Co = colimitation model). Numerical exploration of the colimitation model was done with randomly generated parameter combinations over the ranges given that satisfied inequality Eq (S2.2) (about 50% of all combinations). Default values used for adaptive dynamics are given in the final column.

Symbol	Meaning	Model	Details	
*Parameters*			**Range**	**Default**
*μ*	Resource input rate	SR	≥ 0	N/A
*μ*_*S*_	Siderophore input rate	Co	[0, 1]	0.001
*μ*_*N*_	Nitrogen input rate	Co	[0, 1]	0.9
*ρ*	Resource wash-out rate	Both	[0, 1]	0.1
*α*	Fixation privatization	Both	[0, 1]	0.875
*γ*	Cost to fixation	Both	[0, 1]	0.4
*δ*	Cell death rate	Both	[0.001,0.5]	0.4
*b*	Maximum fixation rate	Co	[0, 2]	N/A
*ℓ*	Fixation half-inhibition	Co	[0, 2]	3
*a*	Maximum nitrogen uptake rate	Co	[0.1,2]	3
*d*	Nitrogen uptake half-saturation	Co	[0, 2]	2
*q*	Maximum siderophore production rate	Co	[0, 2]	1.75
*m*	Siderophore production half-inhibition	Co	[0, 2]	1
*u*	Maximum siderophore uptake rate	Co	[0.1,2]	1
*p*	Siderophore uptake half-saturation	Co	[0, 2]	1.85
*β*	Cost of siderophore production	Co	[0, 1]	0.4
*r*	Maximum cell growth rate	Co	[0.1,2]	2
*k*_*S*_	Strength of colimitation	Co	[0,0.5]	0.1
*c*	Siderophore affinity	Co	[0.1,2]	1.25
*Functions*			**Equation or Figure**	
*g*_*i*_(*R*)	Per capita population growth rate of strain *i* excluding mortality	SR	Eqs (2a) and (2b)	
*f*(*R*)	Resource production rate	SR	n/a	
*U*(*R*)	Resource uptake rate	SR	n/a	
$R_{i}^{*}$	Strain *i*’s resource equilibrium	SR	Fig 1	
$R_{1}^{f}$	Resource concentration where production halts	SR	S1 Fig	
*G*	Invasion growth rate	Both	Eqs (S1.1), (S4.1)	
$f_{R}^{i}\left(R\right)$	Production rate of resource *R* by strain *i*	Co	Eqs (4) and (5); Fig 2a	
*U*_*R*_(*R*)	Rate resource *R* taken up from environment	Co	Eqs (6) and (7); Fig 2a	
*η*_*i*_(*N*)	Rate strain *i* acquires nitrogen	Co	Eq (10)	
G(S,N)	Per capita colimitation growth term	Co	Eq (11); Fig 2c	
*h*_*i*_(*S*)	Half saturation constant for growth on nitrogen	Co	Eq (12)	
S(S,N)	Sensitivity of growth to nitrogen acquisition	Co	Eq (14)	

Cell strain 1 facultatively produces the resource (e.g., fixes nitrogen). Cell strain 2 is a LOF mutant, identical in every way except that it cannot produce the resource. Our assumption that strains differ only in their resource production abilities allows us to explore the scenario in which a LOF mutation arises in a population of producers, and ask whether such a mutation would invade. Both strains take up resource from the extracellular environment to grow, and do so at a rate *U*(*R*). The producing strain also produces resource at rate *f*(*R*), paying a cost of *γ* per unit of resource produced. A proportion *α* of produced resource is privatized (i.e., kept with the producing cell for its own growth benefit), with the other 1 − *α* of the produced resource leaked into the environment. With these assumptions, and because we model the units of resource concentration to map directly onto cell growth, the strain-specific growth functions are

$$\begin{array}{c} g_{1}\left(R\right)=\underset{\begin{array}{c} \text{net}\;\;\text{private}\;\;\text{benefit}\\ \text{of}\;\;\text{production} \end{array}}{\underset{︸}{\left(\alpha -\gamma \right)f\left(R\right)}}+\underset{\text{uptake}}{\underset{︸}{U\left(R\right)}}, \end{array}$$ (2a)

$$\begin{array}{c} g_{2}\left(R\right)=U\left(R\right). \end{array}$$ (2b)

To consider open systems such as oceanic communities, we assume resource enters the system from sources other than leakage from strain 1’s production at rate *μ*, and thus cell populations may persist even in the absence of in situ public goods production. We assume that resources decay (or are washed out of the system) at per capita rate *ρ*, for example as a consequence of layer mixing in oceanic systems. Then, the dynamics of the extracellular resource level are described by

$$\begin{array}{c} \frac{dR}{dt}=\underset{\text{external}\;\text{supply}}{\underset{︸}{\mu }}-\underset{\text{washout}}{\underset{︸}{\rho R}}+\underset{\text{leakage}\;\text{from}\;\text{producers}}{\underset{︸}{\left(1-\alpha \right)f\left(R\right)x_{1}}}-\;\underset{\text{uptake}\;\text{by}\;\text{cells}\;}{\underset{︸}{U\left(R\right)\left(x_{1}+x_{2}\right)}}. \end{array}$$ (3)

To make our results as general as possible, we refrain from specifying the particular forms of the resource production *f*(*R*) and uptake *U*(*R*) functions. However, some constraints are needed to ensure biological realism. All parameters and functions must be non-negative. Since the production rate is expected to decline with increasing resource in the environment, *f*(*R*) is a non-increasing function of *R* (*f*′ (*R*) ≤ 0). We also assume that uptake rate monotonically increases (though it may saturate) with resource concentration, so *U*′(*R*) > 0. Finally, to ensure that the resource can never inhibit producer growth, we assume that $g_{1}^{\prime }\left(R\right)\geq 0$.

**Fig 1:**
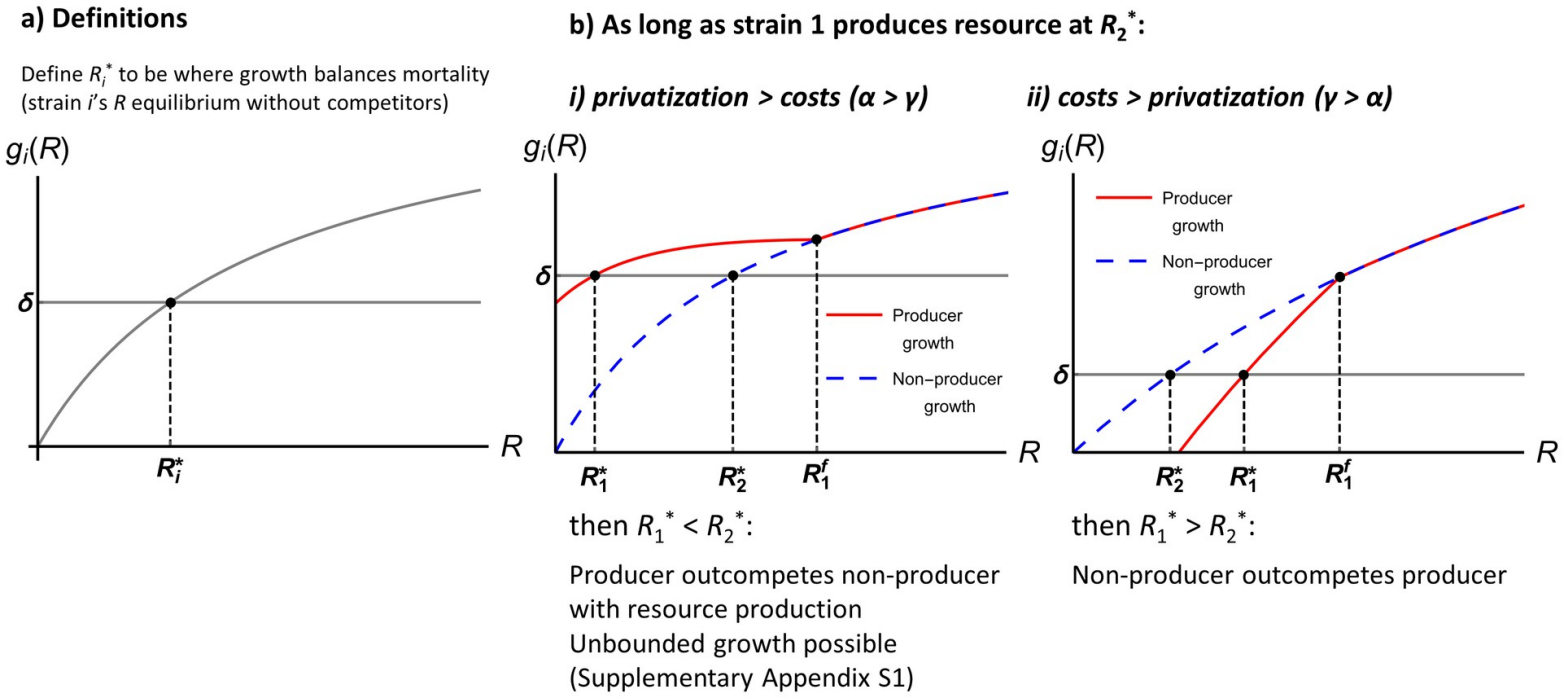
Overview of results from the single-resource model. (a) Graphical representation of single-strain equilibrium $R_{i}^{*}$. (b) The strains’ per capita growth rates excluding external mortality *g*_*i*_ as a function of resource concentration *R*. Note that when growth *g*_*i*_(*R*) balances mortality *δ*, the cell population is at equilibrium. (b) $R_{1}^{f}$ is defined as the resource concentration at which the producer halts production (S1 Appendix). As long as the producer actually produces resources at equilibrium (see S1 Appendix for case where this does not hold), competitive exclusion occurs. i) If privatization is greater than production costs (*α* > *γ*), then the producer outcompetes the non-producer and resource production is maintained. Note that unbounded growth may occur in this case (see S1 Appendix). ii) If production costs are greater than privatization (*γ* > *α*), then the non-producer outcompetes the producer and resource production is lost from the community.

Confirming intuition, we show (S1 Appendix) that Tilman’s R* rule holds in the case of resource production: the consumer with the lowest resource concentration at equilibrium in the absence of competing consumers will competitively exclude consumers with higher resource concentrations at their corresponding equilibria [26, 44, 45]. Thus, to find which strain will win the competition in our model, we must find the relative ordering of their resource concentrations at equilibria in the absence of their competitor ($R_{i}^{*}$; which occurs when growth *g*_*i*_(*R*) balances external mortality *δ*, see Fig 1a for schematic).

### Results: Single resource model

So long as the producer still produces resource at the non-producer’s resource equilibrium ($R_{2}^{*}$), competitive exclusion occurs (see S1 Appendix for the case where the producer does not produce resource at the non-producer’s equilibrium). If the producer never completely halts resource production (for example, if *f*(*R*) asymptotically approaches 0), this is the only possible case. The winning strain depends on the relative strengths of privatization and cost of resource production. If private benefits outweigh the costs of production (*α* > *γ*), then the producer excludes the non-producer ($R_{1}^{*}<R_{2}^{*}$; Fig 1bi). However, if costs of production outweigh private benefits (*γ* > *α*), then the non-producer excludes the producer ($R_{1}^{*}>R_{2}^{*}$; Fig 1bii).

The R* rule implies that when the producer outcompetes the non-producer, resource production has the net effect of leading to a *lower* extracellular resource concentration at equilibrium due to higher cell population densities (analogous to the rebound effect in economics; [46]). This makes clear the importance of re-evaluating past results in a resource-explicit setting. More producers may not lead to more resource (and thus greater benefit to non-producers, as in past phenomenological models): successful producers can actually draw down resources to a point where non-producing is no longer viable.

In summary, our analysis confirms that the central role of privatization documented for public services [22], and in other models that do not explicitly consider the extracellular environment (e.g., [20]), can be extended to resource-explicit models of public consumables. Resource production can stably occur in a well-mixed system, but it requires private benefits to the producer to outweigh the costs of production. We consider evolution of production rates using the framework of adaptive dynamics in S1 Appendix and find that higher production rates will evolve under the same conditions as ecological stability (*α* > *γ*).

## Case study: Colimiting resources in marine cyanobacteria

Now that we have presented the general modeling approach and established that privatization remains key to public goods production when we account for the extracellular environment, we ask how these insights apply to more realistic scenarios. In particular, we focus on the presence of multiple, colimiting resources that can be produced or taken up in the environment, since nutrient colimitation is increasingly understood to shape microbial communities in globally important systems such as the oligotrophic open ocean and many other aquatic systems [42, 43].

In those systems, low levels of multiple nutrients (including both micronutrients like Fe, Co, and Zn and macronutrients like C, N, and P) can jointly limit, or “colimit”, microbial growth. Colimitation can arise (i) between independent pathways, when each pathway requires a resource available at or near limiting concentrations; (ii) within a pathway that can tolerate resource substitution, e.g., of sufficiently similar trace metals in metalloenzymes (enzymes that have metal active sites or co-factors); or (iii) between pathways that each rely on a resource made available by the other pathway [43]. In this third type of colimitation, biochemical dependence between the pathways could couple their evolutionary trajectories, giving strains that either retain both or lose both pathways an advantage over strains that retain just one. Alternatively, the availability of public goods may allow pathways to be lost independently. Understanding the ecology and evolution of consumer-resource systems with biologically dependent colimitation is a major goal of our study.

**Fig 2:**
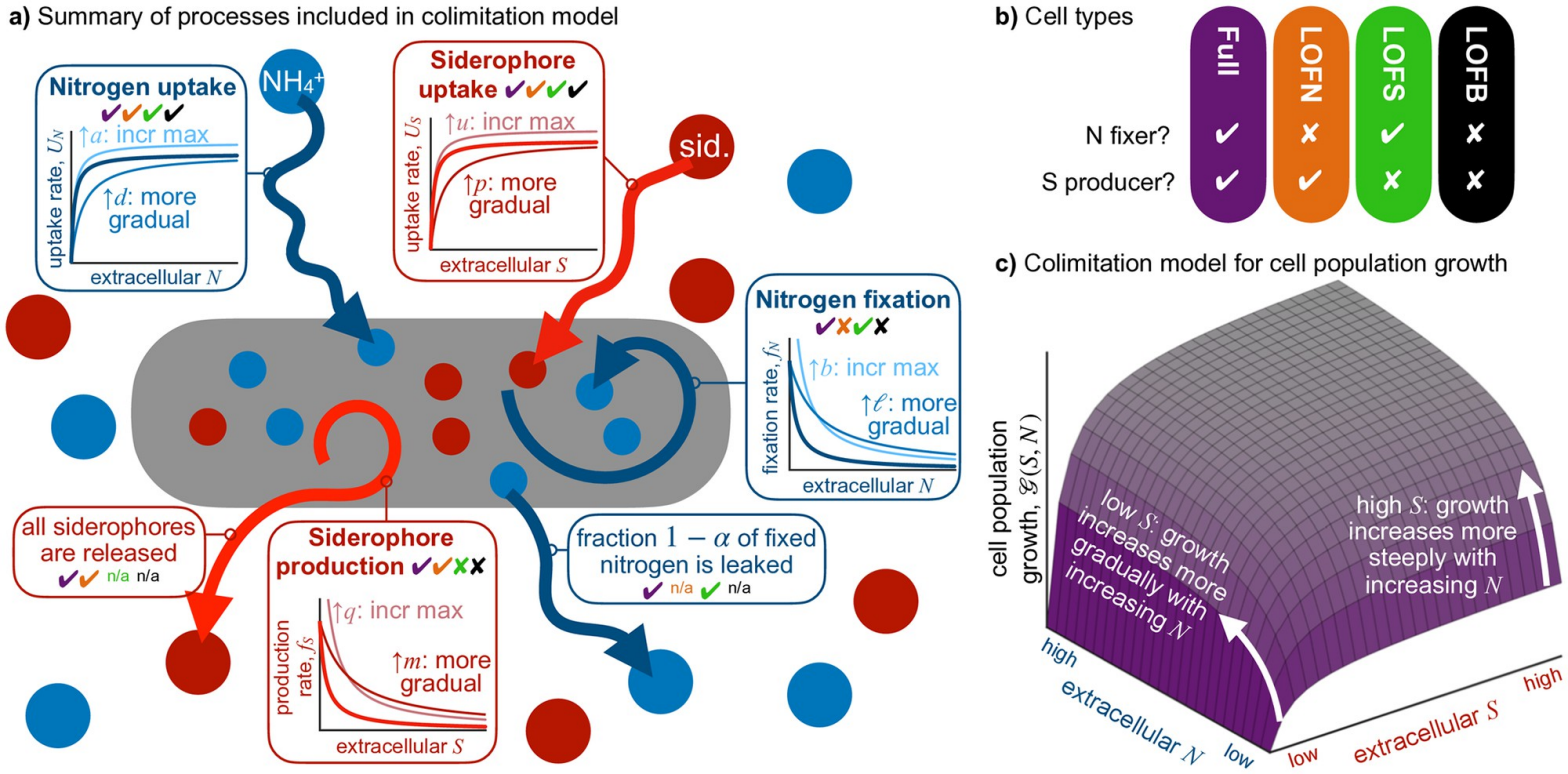
A broad overview of the colimitation model system. (a) We model cell populations that uptake, and may facultatively produce, nitrogen and siderophores at rates determined by the extracellular concentrations of these resources. The effects of increasing each production/uptake parameter, relative to the thickest curve, is shown. (b) We consider four different strains (functions performed by these strains marked with checks in (a), functions not performed marked with x’s): Full (fixes nitrogen and produces siderophores), LOFN (a LOF fixation mutant that only produces siderophores), LOFS (a LOF siderophore mutant that only fixes nitrogen), and LOFB (a LOF mutant that neither fixes nitrogen nor produces siderophores). (c) Because extracellular resource concentrations govern resource uptake and, when applicable, production, the cell population growth rate is also a function of extracellular resource concentrations. According to our colimitation model, availability of siderophores mediates the effect of fixed nitrogen on growth.

Here, we focus on public goods production in the context of the colimitation of the biochemically dependent consumables iron and fixed nitrogen (see Fig 2 for overview). Iron acquisition is complicated by the low solubility and bioavailability of Fe(III). For many microbes, iron acquisition is facilitated by siderophores, nitrogen-rich iron-scavenging compounds that are synthesized and released to the environment, then taken back up from this public pool when they have bound and reduced oxidized iron [47–49]. Siderophores enhance iron solubility, increasing uptake despite significant losses to the public pool [50]; these losses may be counteracted by gains from the public pool in dense populations [47], or curtailed by the use of different classes of siderophores (e.g., amphiphiles that are more likely to associate with the producer’s membrane than to diffuse away; [49]). Such membrane-associated siderophores are not fully public goods but still increase external iron solubility [50, 51] and are relatively rare in some parts of the surface ocean [48, 52]. Although a model of diffusive siderophore loss has led to the suggestion that siderophore production is advantageous only in very dense populations [47], analysis of the distribution of siderophore production genes among *Vibrio* isolates shows that large particles tend to harbor cheaters while planktonic growth is positively correlated with siderophore production [18]. This conflict between experiment and a model that considers only the environment suggests that the evolutionary dynamics of siderophore production should be considered in the context of both competitive interactions and the extracellular environment.

The biochemical dependence between siderophore production and nitrogen fixation runs in both directions: not only does production of nitrogen-rich siderophores increase nitrogen demand, but also nitrogen fixation is a heavily iron-dependent process. Cells that do not fix nitrogen are limited to acquiring fixed nitrogen from the public pool. Thus, iron and fixed nitrogen are two biochemically dependent resources that can have asymmetric impacts on cell population growth [43, 53]. An additional asymmetry arises from the different “leakiness” behavior of siderophores and fixed nitrogen. Fixed nitrogen becomes a public good only incidentally, through diffusive loss and secretion of some fraction of downstream metabolites [53, 54]. By contrast, siderophores must be secreted to be active and enhance the overall iron solubility; leaving aside membrane-associated siderophores as a special case, siderophores can be considered a fully public good. Focusing on such freely diffusible siderophores (as opposed to those that are partially privatized) alongside nitrogen fixation allows us to investigate the interaction between colimiting public goods with different positions on the “leakiness” spectrum [32].

As before, motivated by the surface ocean, we assume a perfectly well-mixed external environment in the colimitation model. That is, we deliberately ignore the large-scale structuring of the marine surface environment by diffusional gradients, thermoclines, restratification, and chemical variations [55–57]. Neither do we consider the small-scale structures provided by particulate organic matter, which provides a key microbial habitat for surface-attached cells (e.g., [58]). We instead model a planktonic community in a well-mixed environment for two reasons. First, understanding constraints on planktonic growth is important for understanding the ocean. Planktonic *Pelagibacter* can constitute 50% of the surface ocean microbial community during blooms, and planktonic *Prochlorococcus*, numerically the world’s most abundant phototroph, fixes 4 Gt carbon annually in the surface ocean [30, 59]. Indeed, it was the study of *Prochlorococcus* that first spurred development of the BQH, with the observation that these picocyanobacteria do not encode the key catalase-peroxidase (katG) enzyme required to quench reactive oxygen species [31]. Second, excluding spatial structure from our analysis allows us to ask whether public goods production requires spatial structure or can instead be supported by privatization alone.

### Colimitation model formulation

Our model consists of four state variables: extracellular nitrogen (e.g. biologically available ammonium concentration) *N*, extracellular siderophore concentration *S* (which we assume is synonymous with biologically available iron), and two cell types, where the density of the *i*^th^ type is denoted *x*_*i*_. We note that considering two resources opens up the possibility that two distinct strains may stably coexist [26]. As in the single-resource model, we assume cell strains are identical except for the degree to which they facultatively produce the two resources (Fig 2b), as expected when the loss of producing ability arises through mutation.

We assume that per capita resource production rates (i.e. nitrogen fixation and siderophore production, respectively) of strain *i* decrease monotonically with environmental resource concentration. We use *b*_*i*_ and *q*_*i*_ as the maximum rates of fixation and siderophore production for strain *i*, which occur when no extracellular resources are available (*N* = 0 and *S* = 0), respectively. Mathematically, production rates are given by the functions

$$\begin{array}{cc} f_{N}^{i}\left(N\right) & =\frac{\ell b_{i}}{\ell +N} \end{array}$$ (4)

$$\begin{array}{cc} f_{S}^{i}\left(S\right) & =\frac{mq_{i}}{m+S}, \end{array}$$ (5)

that asymptotically approach 0 as extracellular resource concentrations become large (Fig 2a). The parameters *ℓ* and *m* are the resource concentrations at which production is half its maximum (half-inhibition constants): smaller values indicate that producers facultatively halt production under lower external resource concentrations. Conversely, we assume that per capita nitrogen and siderophore uptake rates increase monotonically with resource concentration to maxima *a* and *u*, respectively, according to a type II function (a saturating curve also known as a Michaelis-Menten function), where *d* and *p* are the half-saturation constants (resource concentrations at which resource uptake is half its maximum) for fixed nitrogen and siderophores, respectively (Fig 2a). Mathematically, uptake rates are given by

$$\begin{array}{cc} U_{N}\left(N\right) & =\frac{aN}{d+N} \end{array}$$ (6)

$$\begin{array}{cc} U_{S}\left(S\right) & =\frac{uS}{p+S}. \end{array}$$ (7)

We assume that nitrogen fixed by a given cell is (partially) privatized by the cell [54]. In particular, a proportion *α* of fixed nitrogen remains with the cell (to be used directly in cell growth) and is not released into the environment, while the remaining 1 − *α* is leaked (Fig 2a). Finally, we assume that each resource molecule decays (or is washed out of the system) at rate *ρ* and that nitrogen and siderophores enter the system from external sources at rates *μ*_*N*_ and *μ*_*S*_, respectively. Thus, the dynamics of resource concentration are

$$\begin{array}{cc} \frac{dN}{dt} & =\mu_{N}-\rho N+\sum_{i=1}^{n}x_{i}(\left(1-\alpha \right)f_{N}^{i}\left(N\right)-U_{N}\left(N\right)) \end{array}$$ (8)

$$\begin{array}{cc} \frac{dS}{dt} & =\underset{\begin{array}{l} \text{external}\\ \text{supply} \end{array}}{\underset{︸}{\mu_{S}}}-\underset{\text{washout}}{\underset{︸}{\rho S}}+\underset{\begin{array}{l} \text{total}\;\;\text{(all}\;\;\text{strains)}\;\;\text{leaked}\\ \;\;\text{production}\;\;\text{minus}\;\;\text{uptake} \end{array}}{\underset{︸}{\sum_{i=1}^{n}x_{i}\left(f_{S}^{i}\left(S\right)-U_{S}\left(S\right)\right)}}, \end{array}$$ (9)

where *n* is the number of cell strains. Note that in the absence of any consumers (*x*_*i*_ = 0, for all *i*), the resource concentration equilibrates at nitrogen and siderophore concentrations of *μ*_*N*_/*ρ* and *μ*_*S*_/*ρ*, respectively. These compound parameters are commonly referred to as “supply points”, and characterize the abiotic properties of the local environment. As we will see, these abiotic properties are important drivers of eco-evolutionary outcomes. Because *μ*_*N*_ and *μ*_*S*_ can be varied independently, our assumption that both resources have the same washout rate does not lead to any loss of generality in supply points. Further, this assumption facilitates the interpretation of our graphical approach [28] and matches the biologically relevant situation where decay is solely a result of convection.

All that remains is to determine the dynamics of the cell strains. While the rate at which cells obtain siderophores is simply *U*_*S*_ since siderophores are completely public in our model, the rate at which cells obtain nitrogen is complicated by privatization. Cells obtain nitrogen through both privatized production ($\alpha f_{N}^{i}$) and uptake from the environment (*U*_*N*_) such that the total nitrogen acquisition rate is

$$\begin{array}{c} \eta_{i}\left(N\right)=\alpha f_{N}^{i}\left(N\right)+U_{N}\left(N\right). \end{array}$$ (10)

Cells decrease nitrogen fixation, $f_{N}^{i}(N)$, and increase nitrogen uptake, *U*_*N*_(*N*), in response to increasing environmental nitrogen concentration per Eqs (4) and (6). For realism, we constrain these responses to prevent total nitrogen acquisition, *η*_*i*_(*N*), from decreasing with increasing environmental fixed nitrogen availability (see S2 Appendix and Eq (S2.2) for details), consistent with observations of nitrogenase repression, nitrogen drawdown, and diazotroph growth under increasing fixed N in chemostat culture [60].

We follow the model of [43] based on [61] for biologically-dependent (type III) colimitation, where the ability to convert fixed nitrogen into biomass, $G_{i}\left(N,S\right)$, is dependent upon the acquisition of siderophores (iron) (Fig 2c). Cell type *i*’s population growth rate increases in proportion to its rate of nitrogen acquisition, *η*_*i*_(*N*), when nitrogen is scarce, but in increasingly nitrogen-rich settings cell growth saturates to a maximum rate of *r*,

$$\begin{array}{c} G_{i}\left(S,N\right)=\frac{r\eta_{i}\left(N\right)}{\eta_{i}\left(N\right)+h_{i}\left(S\right)}. \end{array}$$ (11)

Colimitation occurs because the half-saturation level, *h*_*i*_(*S*), of this growth function depends on siderophore acquisition. From [61], biochemically dependent colimitation is characterized by a half-saturation function of the form

$$\begin{array}{c} h_{i}\left(S\right)=\frac{r}{c}(\frac{U_{S}\left(S\right)+k_{S}}{U_{S}\left(S\right)}), \end{array}$$ (12)

in which *U*_*S*_(*S*) is the siderophore uptake rate from Eq (7). With unlimited uptake of siderophores, the half-saturation constant is $\frac{r}{c}$, meaning that population growth occurs at half its maximum rate of *r* when nitrogen is obtained at rate $\eta_{i}\left(N\right)=\frac{r}{c}$. Lower siderophore levels decrease population growth by raising the half-saturation constant, to $2\frac{r}{c}$ when siderophore uptake *U*_*S*_(*S*) = *k*_*S*_, and to infinity when there is no siderophore uptake.

In addition to colimited growth (Eq 11), we assume a population growth cost of *γ* and *β* per unit rate of fixation and siderophore production, respectively, and that cells die or are washed out of the population at per capita rate *δ*. Thus, the dynamics of strains *i* = 1, …, *n* are

$$\begin{array}{c} \frac{dx_{i}}{dt}=x_{i}\left(\underset{\begin{array}{l} \text{growth}\;\;\text{at}\;\;\text{current}\\ \text{resource}\;\;\text{level} \end{array}}{\underset{︸}{G_{i}\left(S,N\right)}}-\underset{\text{N}}{\underset{︸}{\gamma f_{N}^{i}\left(N\right)}}\;\text{fixation}\;\text{cost}-\underset{\begin{array}{l} \text{S}\;\;\text{production}\\ \text{cost} \end{array}}{\underset{︸}{\beta f_{S}^{i}\left(S\right)}}-\underset{\begin{array}{l} \text{death/}\\ \text{washout} \end{array}}{\underset{︸}{\delta }}\right). \end{array}$$ (13)

Collectively, Eqs (8), (9) and (13) make up the dynamical system that we study. All model parameters are summarized in Table 1.

### Pairwise competition

We focus our analysis of this case study of marine cyanobacteria on the following questions. Does the ability to produce both resources (the “fully functional” strain) carry an advantage over the various LOF mutations and thus permit the maintenance of public goods production? Does colimitation affect which resources can be stably produced? And how do environmental conditions alter competitive outcomes?

#### Graphical analysis: The role of the environment

The standard approach for studying multi-resource consumer-resource models is a graphical analysis of the system’s zero net growth isoclines (ZNGIs)—the set of resource concentrations for which a given strain neither grows nor shrinks that defines the boundary between population growth and decline. In addition to showing the environmental conditions that permit growth, ZNGIs also allow one to graphically determine the outcome of competition between multiple strains [26, 45, 62]. Box 1 provides an overview of the graphical approach and the information that it provides. We used the graphical approach to assess pairwise competition between each pair of strains, supplementing conclusions with analytical results about competitive dominance in the limits of a single resource in excess.

**Box 1: Graphical analysis of consumer-resource models**

**Fig 3:**
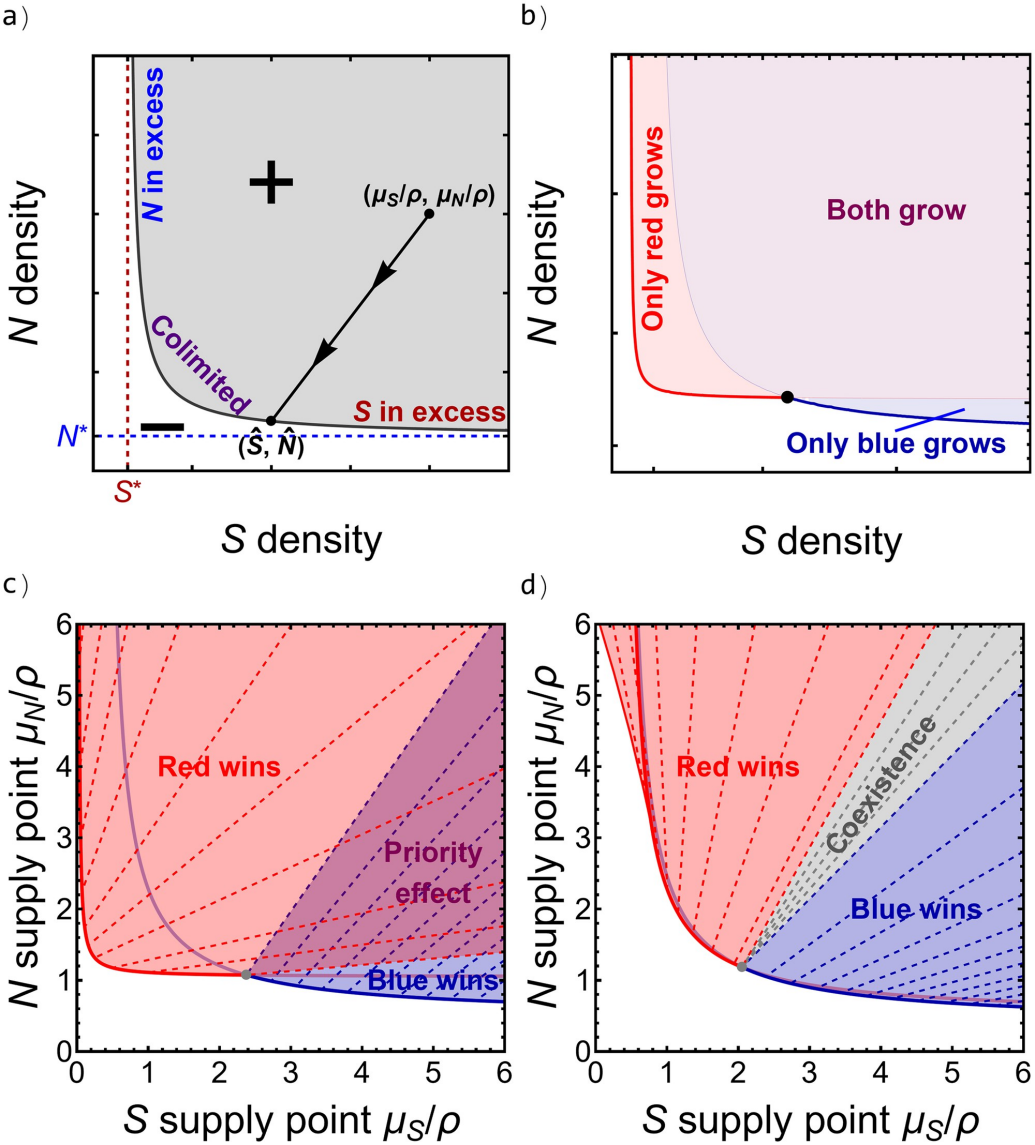
Graphical analysis of consumer-resource models (see Box 1 for details). (a) Illustration of a zero net growth isocline (ZNGI, gray curve) for a cell population as a function of resource availabilities. The shaded region marked with a “+” denotes the resource levels for which the strain can grow. Whether *S*, *N*, or both resources are limiting depends on the resource availabilities relative to the minimum individual concentrations required for growth (S*, N*). Impact vectors (the thick line with arrows is one example) map resource supplies onto equilibrium resource concentrations. (b) Drawing ZNGIs for two different strains allows for an assessment of the conditions in which both (purple) or only one (red or blue) strain can grow. (c) The outcome of competition between two strains also requires knowledge of how they influence the environment (their impact vectors; dashed lines). Here, coexistence (necessarily at the intersection of the two ZNGIs) is unstable (a priority effect) because each strain draws down the resource that its competitor most needs to grow more than the resource that it itself most needs to grow. (d) Stable coexistence happens when each strain draws down the resource that it most needs to grow more than the resource that its competitor most needs to grow.

A convenient graphical tool for characterizing the outcome of resource competition (coexistence, competitive exclusion, priority effects) along varying supplies of the two resources involves two steps: 1) an isocline analysis and 2) a supply point mapping. For the isocline analysis, one draws the *zero net growth isocline* (ZNGI; gray line in Fig 3a) for the cell strain in question. This is the set of resource concentrations (*S*, *N*) at which the cell population is at equilibrium. The gray region above the ZNGI labeled with “+” is the range of resource concentrations at which the cell population can grow (its fundamental niche), while the white region below the ZNGI labeled with “−” is the range of resource concentrations at which the cell population declines. The shape of our ZNGI is characteristic of interactive-essential resources (sensu [35]), which have three regimes. When N is in excess for growth (*N* → ∞), or equivalently, siderophores are limiting, the ZGNI has a vertical asymptote (S*) giving the minimum siderophore concentration required for growth. Similarly, the horizontal asymptote (N*) gives the minimum N concentration required for growth. The curved portion of the ZNGI connecting these two regimes represents colimitation by the two resources.

Plotting ZNGIs for two different cell strains allows for an assessment of competitive outcomes as environmental conditions change (Fig 3b). First, any coexistence equilibrium must, by definition, occur at the intersection of two ZNGIs. Second, when the red ZNGI falls below the blue ZNGI (for example), resource concentrations between the two lines correspond to regions where the red species, but not the blue species, can grow. To determine how this maps onto equilibrium competitive outcomes, one must also determine how consumers change resource concentrations from their *supply points* (the equilibrium resource concentrations in the absence of any cell population, *μ*_*S*_/*ρ*, *μ*_*N*_/*ρ*). *Impact vectors* (black line in Fig 3a; dashed lines in Fig 3c and 3d) map supply points onto equilibrium resource concentrations: when introducing a cell strain into an environment characterized by a supply point (*μ*_*S*_/*ρ*, *μ*_*N*_/*ρ*), resource levels will change (in this example, be drawn down) as both resources and cells equilibrate to a point ($\hat{S}$, $\hat{N}$) on the ZNGI.

Thus, drawing impact vectors and ZNGIs for multiple strains as a function of supply points provides a bifurcation diagram that maps environmental conditions (supply points) onto equilibrium outcomes [62]. Impact vectors at the coexistence equilibrium are especially useful because they determine whether stable coexistence or a priority effect (competitive exclusion with the winner determined by initial conditions) will occur. The region delimited by a strain’s ZNGI and its competitor’s impact vector at the coexistence equilibrium is its realized niche. Thus, for example, resource levels from which red impact vectors trace onto the red ZNGI will result in the red strain excluding the blue strain. If each species impacts the resource that limits its competitor more than itself at equilibrium (Fig 3c), then a priority effect will arise. If the opposite is true, then coexistence is stable (Fig 3d).

#### Sensitivity analysis: Viability and invasibility

To better assess a broad range of parameters (and because fully-factorial analyses quickly become intractable for a 18-parameter model), we produced parameter combinations by randomly drawing parameter values from the uniform distributions given in Table 1 and keeping 4 million parameter combinations that satisfied the constraint from Eq (S2.2). Note that the constraint leads to some correlations between used parameters and thus the realized distribution is not the same as the uniform distribution from the table. These parameters provide 4 million putative fully functional strains, and allowed us to create 4 million of each of the other strains (LOF mutants who fix no nitrogen, produce no siderophores, or perform neither function) by setting the production parameters (*b* and/or *q*) to zero.

For each randomly generated parameter combination, we considered four residents (Fig 2b): the fully-functional strain (Full; able to produce both resources), a fixation LOF mutant (LOFN; unable to fix N, *b*_*R*_ = 0, where the subscript *R* denotes resident), a siderophore LOF mutant (LOFS; unable to produce siderophores, *q*_*R*_ = 0), and a dual LOF mutant (LOFB; unable to produce either resource, *b*_*R*_ = *q*_*R*_ = 0). First, we sought to determine how often each resident type was viable and how various parameters affected their viability. Taking one focal parameter at a time, we sorted all 4 million random parameter combinations according to their value for the focal parameter. We then divided the focal parameter’s range into 100 evenly spaced intervals. Within each interval, we calculated the proportion of parameter combinations that were viable (S2 Fig). We assessed viability through numerical integration for 10^5^ time units from initial conditions *N*(0) = *S*(0) = *x*_*R*_(0) = 0.3, defining the final values of *N*, *S*, and *x*_*R*_ as N*, S*, and $x_{R}^{*}$, respectively.

Second, we sought to determine how various parameters affected invasibility of each resident by a given invader. For each parameter combination, we eliminated non-viable resident strains, i.e., those that went extinct (had final density < 10^−8^) in the viability assessment. Once more, we began one parameter at a time and divided the entire range of the focal parameter into 100 evenly spaced intervals. Then we computed the probability that an invasion was successful within each interval for each type of invader (i.e. the Full, LOFN, LOFS, or LOFB strains generated from the same parameter combination as the resident) (S3 Fig), leading to six possible cases of pairwise competition between the four strains (Full vs. LOFN, Full vs. LOFS, Full vs. LOFB, LOFN vs. LOFS, LOFN vs. LOFB, and LOFS vs. LOFB). We assessed successful invasion by calculating the invasion growth rate at the resident’s equilibrium (N* and S*). Importantly, this procedure results in correlations between the various parameters. For example, high death rates are unlikely to be viable unless the maximum growth rate is also high. Thus, whenever we compute intervals for high death rates we are indirectly selecting parameter sets with high growth rates. Such correlations are inevitable, but it is important to keep in mind that specifying the range of a single parameter does not result in an independent sample of all other parameters.

To draw more focused conclusions, we consider two particularly important parameters: privatization of fixed nitrogen, *α*, and cost of nitrogen fixation, *γ*. We compute the probability of successful invasion (for each specific resident and invader combination) for different privatization, *α*, and fixation costs, *γ*. To do this, we divide (*α*, *γ*) parameter space into a 50 × 50 lattice and choose all viable parameter combinations (from the 4 million generated) that fall within a distance of 0.05 to each lattice point (the disk of radius 0.05 centered at the lattice point). Within each disk, we compute the probability that the invasion is successful.

#### Results: Colimitation model sensitivity analysis and the graphical approach

**Fig 4:**
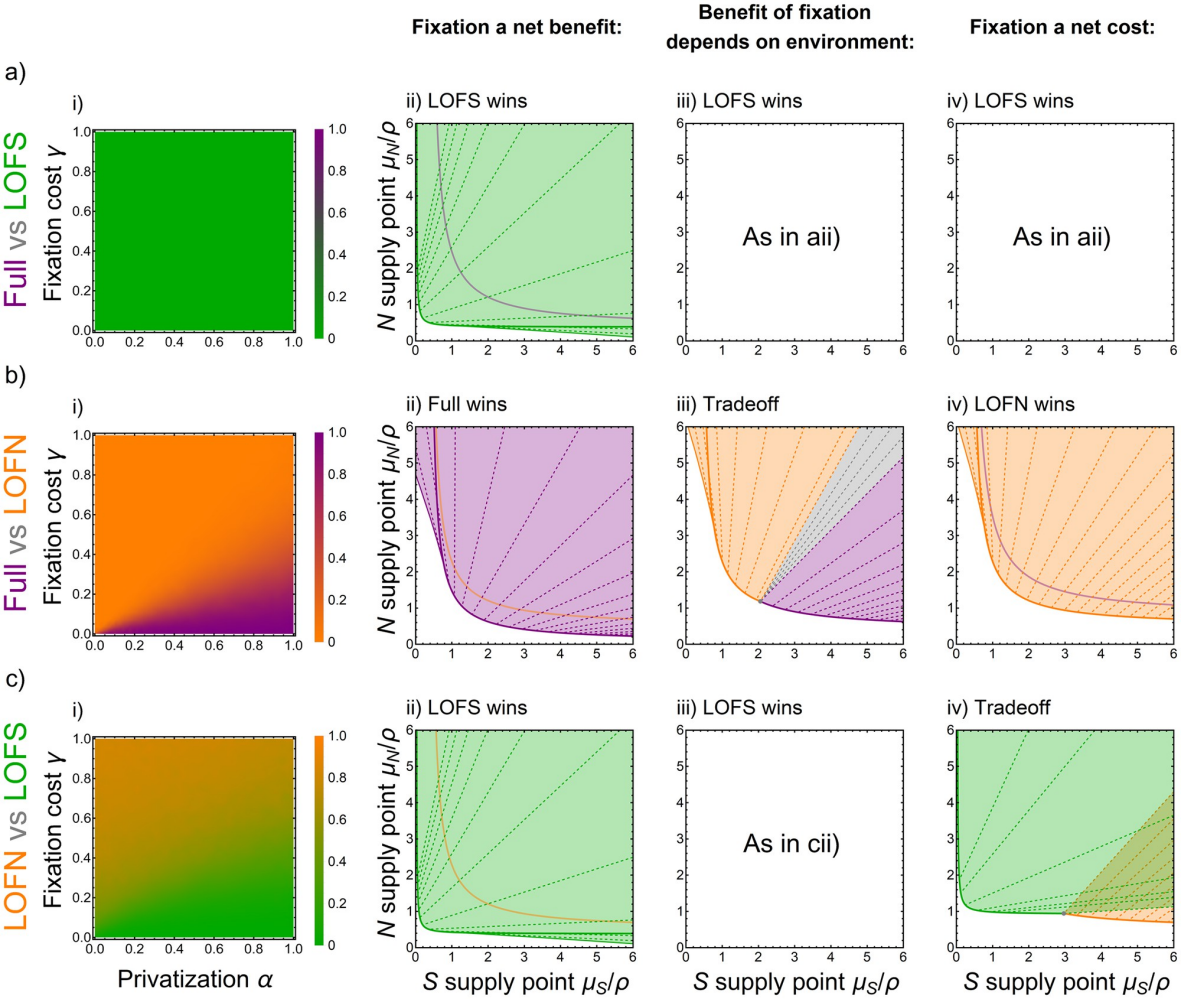
Sensitivity analysis and ZNGIs for the role of privatization *α* and cost of fixation *γ* for three pairwise competition scenarios (rows). The first column of each row (i) shows the probability that the second strain listed ((a) LOFS, (b) LOFN, (c) LOFS) successfully invades the first strain listed ((a,b) Full, (c) LOFN). Subsequent columns of each row plot outcomes of competition against the external environment (see Box 1 for explanation) when: (ii) fixation is beneficial even when N is in excess such that siderophores are limiting, i.e. $\alpha >\gamma /S(S^{*},\infty )$; (iii) fixation is beneficial when N is limiting but costly when siderophores are limiting, i.e. $\gamma /S(S^{*},\infty )>\alpha >\gamma /S(\infty ,N^{*})$; (iv) fixation is costly even when N is limiting, i.e. $\alpha <\gamma /S(\infty ,N^{*})$. (a) Full vs. LOFS. i) Regardless of parameters, LOFS can successfully invade Full’s resident equilibrium ii-iv) because the ZNGI for LOFS always falls below the ZNGI for Full (siderophore production is always a net cost). (b) Full vs. LOFN. i) Full can resist invasion from LOFN if fixation is private (high *α*) and not very costly to produce (low *γ*). ii) If fixation is always a net benefit, then Full always wins. iii) If the benefit of fixation depends upon the environment, then either strain could win or they could coexist (with the outcome depending on environmental resource supplies). iv) If fixation is always a net cost, then LOFN always wins. (c) LOFN vs. LOFS. i) LOFS can invade LOFN if fixation is private (high *α*) and not very costly to produce (low *γ*). ii) If fixation is a benefit when siderophores are limiting, then LOFS outcompetes LOFN regardless of environmental conditions. iii) If the benefit of fixation depends upon the environment, then LOFS either always outcompetes LOFN (shown here) or two coexistence equilibria arise (shown in S5 Fig), with the latter case arising if fixation is more costly than siderophore production moving away from S*. iv) If fixation is costly when nitrogen is limiting, then LOFN wins in N-limited regimes and LOFS wins in siderophore-limited regimes, with a priority effect in regions of overlap.

We begin by analyzing the straightforward case of siderophore production. Producing siderophores reduces a strain’s viability (Table 2). Strains that produce siderophores are the only ones to suffer the production cost *β*, and for the fully public siderophores considered here, siderophore-producers are no more likely than non-producers to take up siderophores from the well-mixed environment. The per capita growth rate of a strain that produces siderophores is always lower than that of a strain that does not produce siderophores and the difference in their growth rates is exactly the siderophore production cost. Then, if all else is equal, a strain that produces siderophores (a fully public consumable) cannot outcompete a strain that has lost this function, regardless of parameters and environmental conditions. Two of our six cases of pairwise competition illustrate this result (Full vs LOFS; LOFN vs LOFB). Indeed, LOFS excludes Full and LOFB excludes LOFN regardless of parameter combinations, resident strain, degree of privatization, and fixation costs, since the producer’s ZNGI always falls below that of the non-producer (Fig 4a; S4a Fig; Table 2).

**Table 2:** Viability and competitive ability of each of our four strains (each column corresponds to a different resident). The first row of the body (‘% Viable’) shows the percentage of parameter combinations for which each resident type was viable. The remaining rows show the percentage of parameter combinations for which the invasion was successful (conditioned on resident viability; left-hand column indicates which strain was the invader). Abbreviations in Fig 2b.

	Resident			
	Full	LOFN	LOFS	LOFB
**% Viable**	34.7	44.7	48.4	58.1
**vs Full invader**	-	15.9	0.0	3.0
**vs LOFN invader**	84.5	-	30.5	0.0
**vs LOFS invader**	100.0	48.3	-	13.9
**vs LOFB invader**	96.7	100.0	85.6	-

Partial privatization makes the case of nitrogen fixation far richer. Analytical results can be found in limiting cases where one of the resources is in excess. These limiting cases help us understand the more complicated case of colimitation, so we present them first. The limiting case of siderophores in excess (*S* → ∞) occurs where the ZNGI approaches a horizontal asymptote at N*, the minimal nitrogen concentration needed to grow (see Box 1). The second limiting case, N in excess, is the vertical asymptote as *N* → ∞ at S*, the minimal concentration of siderophores needed for growth. We find that a strain’s minimum siderophore requirement S* is determined only by whether or not it produces siderophores: consistent with the result above, siderophore production always increases a strain’s S*, making it a weaker competitor under siderophore limitation (S2 Appendix).

Likewise, a strain’s minimum nitrogen requirement N* depends only on whether or not it fixes nitrogen. We can acquire analytical results for whether or not fixation is beneficial in the neighborhood of N* with siderophores in excess, and in the neighborhood of S* with N in excess. For N* we can assess this directly by determining whether increasing fixation *b*_*i*_ decreases N* ($\frac{\partial N^{*}}{\partial b_{i}}<0$) and thus is favorable. In particular, increased fixation is beneficial (lowers N*) when the realized benefits (the private benefits *α* weighted by the sensitivity of growth to increased availability of fixed nitrogen; direct benefits do not accrue if growth has already saturated) are larger than the direct costs *γ* (see S6 Fig for the effect of specific parameters). Notably, fixation is never beneficial without private benefits *α*. More formally, we define the sensitivity of growth to nitrogen acquisition as

$$\begin{array}{c} S\left(S^{\prime },N^{\prime }\right)=\frac{\partial G}{\partial \eta_{i}}{|}_{\begin{array}{l} S=S^{\prime }\\ N=N^{\prime } \end{array}} \end{array}$$ (14)

and find a lower bound on privatization for fixation to be beneficial under nitrogen limitation in S2 Appendix of

$$\begin{array}{c} \alpha >\frac{\gamma }{S(\infty ,N^{*})}. \end{array}$$ (15)

Under nitrogen limitation, goods that are “more public” than given by Eq (15) are not expected to be sustained through ecological competition. The denominator of the right-hand side demonstrates that how public produced resources can be while still benefiting the producer depends critically on the external environment, which shapes the sensitivity of cell population growth to the produced resource. Of special interest is whether fixation is beneficial when *b*_*i*_ = 0 (i.e., is fixation favored in a population that does not fix?). Simplifying inequality (15) when *b*_*i*_ = 0 shows that fixation is most likely to evolve from its absence for large maximum growth rates *r* and low death rates *δ*. The evolution of fixation also requires that colimitation not be too strong (low *k*_*S*_). Mathematically, fixation is beneficial in the neighborhood of N* with *b*_*i*_ = 0 when

$$\begin{array}{c} \alpha >\frac{1+k_{S}}{c}\frac{r\gamma }{r-\delta }. \end{array}$$ (16)

Although fixation does not influence the case of complete siderophore limitation, where *N* → ∞ and *S* = S*, we can approximate to the first order the ZNGI about S* to find when fixation is beneficial in this limit (S2 Appendix). The qualitative conclusions described above hold, and the lower bound of privatization for fixation to be beneficial under siderophore-limitation is

$$\begin{array}{c} \alpha >\frac{\gamma }{S(S^{*},\infty )}. \end{array}$$ (17)

Due to inequality (S2.2), the benefits of increasing fixation under siderophore limitation are lower relative to the case of N limitation (formally, $S\left(S^{*},\infty \right)<S\left(\infty ,N^{*}\right)$) such that inequality (15) is satisfied whenever inequality (17) is satisfied. To summarize, the N-fixing strain can be (i) better when both N- and siderophore-limited, thus better than the non-fixer for any supply point ($\alpha >\frac{\gamma }{S(S^{*},\infty )}$), and we say that *fixation is a net benefit*; (ii) better when N-limited, but worse when siderophore-limited, thus trading off with the non-fixer at intermediate resource availabilities ($\frac{\gamma }{S(\infty ,N^{*})}<\alpha <\frac{\gamma }{S(S^{*},\infty )}$), and we say that *the benefit of fixation depends on the environment*; or (iii) worse when both N- and siderophore-limited, thus worse than the non-fixer for any supply point ($\alpha <\frac{\gamma }{S(\infty ,N^{*})}$), and we say that *fixation is a net cost*. These conditions make clear that changing environmental conditions may alter the competitive hierarchy between two strains.

The conditions for which fixation is a net benefit also allow us to understand the outcomes of pairwise competition outside of these *N* → ∞ and *S* → ∞ limiting cases. There are two competitive scenarios in which the only difference between strains is the ability to fix N (Full vs LOFN; LOFS vs LOFB). In both cases, either strain may win the competition (Table 2). Broadly, inequalities (15) and (17) show that there are two ways to make nitrogen fixation more favorable: 1) by making fixation more necessary through changing environmental conditions (increasing S by changing external resource concentrations) and 2) by making the process of fixation more beneficial through changing its costs (*γ*) or benefits (*α*). First, when growth is primarily N-limited (horizontal asymptote of the ZNGIs), growth is expected to strongly respond to an increase in N acquisition through fixation. This means that environmental conditions leading to N limitation favor N-fixing strains (Fig 4biii; S4biii Fig). If fixation is a net benefit even under siderophore limitation (inequality (17) is satisfied), then the N-fixing strain will always win the competition (Fig 4bii; S4bii Fig). In contrast, if fixation is a net cost even under N limitation (inequality (15) is not satisfied), then the non-fixing strain will always win the competition (Fig 4biv; S4biv Fig). Second, N fixation can only be favored if fixation is sufficiently private and not too costly (Fig 4bi; S4bi Fig).

We have assessed pairwise competition in four of our six possibilities; the two remaining cases involve strains that differ in both kinds of production (Full vs LOFB; LOFN vs LOFS) and thus allow us to assess possible interactions between the production of colimiting resources. We first consider competing Full against LOFB. The winner depends upon whether the net benefit of fixation (if it exists; inequality (15)) outweighs the net cost of siderophore production. The first way to affect this competition is through environmental conditions: fixing nitrogen is more beneficial when cell population growth is limited by lack of nitrogen availability (S4cii–S4ciii Fig). The second is by increasing the net benefit of nitrogen fixation by making fixation more private or less costly (S4ci Fig). Importantly, because siderophore production can only increase a strain’s S*, there are limits to the conditions under which Full can outcompete LOFB: in a sufficiently strong regime of *S* limitation, LOFB will always outcompete Full (i.e., the LOFB ZNGI will always fall outside of the Full ZNGI along the vertical asymptote; S4c Fig). Given this, as expected, we find that LOFB is more likely to outcompete Full across parameter sets than vice versa (Table 2). The fact that Full sometimes wins this competition demonstrates that a fully-public good can be stably produced if its production is subsidized by the production of a partially privatized good. This occurs when the private benefit of producing a partially privatized good outweighs the cost of producing a fully public good.

Likewise, there are limits to the conditions under which LOFN can outcompete LOFS. The winner depends upon the conditions under which fixation is a net benefit. Again, siderophore production can only increase a strain’s S*, so LOFS will always outcompete LOFN given a sufficiently strong regime of siderophore limitation (Fig 4civ). Whether or not LOFN outcompetes LOFS under N limitation depends upon whether fixation is a net cost or net benefit in this regime (inequality (15)). If fixation is a net cost, then LOFN will outcompete LOFS under N limitation (Fig 4civ). If fixation is a net benefit, then LOFS can outcompete LOFN regardless of environmental conditions (Fig 4cii). If the benefit of fixation depends on the environment, then there are two possibilities. First, if siderophore production is costlier than fixation moving away from S*, then LOFS will always outcompete LOFN (Fig 4ciii). Second, if fixation is costlier than siderophore production moving away from S*, then LOFN can begin to outcompete LOFS. As fixation becomes beneficial moving towards N*, their competitive abilities again switch and LOFS outcompetes LOFN, creating a second coexistence equilibrium (S5 Fig). Overall, whether or not LOFN can outcompete LOFS when the benefit of fixation depends on the environment is determined by the relative costs of siderophore production and fixation in the neighborhood of S*. Across parameter sets, LOFS is more likely to outcompete LOFN (Table 2). As shown previously, making fixation more private and less costly favors the LOFS strain in this competition (Fig 4ci). Still, the fact that LOFN sometimes outcompetes LOFS demonstrates that partially privatized resources can be costlier than fully public resources under some conditions.

We have focused primarily on competitive abilities (N* and S*) without considering the effect of the strains on the environment. Assessing such feedbacks comes naturally in our resource-explicit approach, where the impact of cell populations on the environment determines the stability of coexistence equilibria. Notably, coexistence equilibria are relatively uncommon in our model (S1 Table). We find that the strains’ relative impact, characterized by the order of impact vectors at the coexistence equilibrium (see Box 1), is predictable (S3 Appendix). Moving counterclockwise from the negative x-axis, one always passes through the impact vectors of LOFN, then LOFB, then LOFS. The impact vector for the fully-functional strain is less constrained, but can be positioned relative to that of LOFN and LOFS based on whether it is a net producer or consumer of each resource at equilibrium (S3 Appendix). Due to general conclusions about whether fixation is beneficial in the limit of siderophore and N limitation (inequalities (15) and (17)), coexistence between LOFS and LOFB is always stable. Coexistence between LOFN and LOFS is always unstable (a priority effect) if there is one coexistence equilibrium (Fig 4ciii). If there are two coexistence equilibria (S5 Fig), then the equilibrium closer to S* is unstable and stable coexistence occurs at the equilibrium closer to N*. As long as siderophore producers are net consumers at equilibrium, coexistence between Full and LOFN is always stable. Both stable and unstable coexistence are possible between Full and LOFB. Results on the prevalence of coexistence equilibria and their stability are summarized in S1 Table. Overall, these results demonstrate that partial privatization does not necessarily lead to the negative frequency dependence necessary to stabilize coexistence. Either environmental conditions or the effect of cell strains on the environment may still prevent coexistence even when the R*’s for the two resources trade-off. As we have seen here, there are many more ways to have exclusion than coexistence in such systems (Fig 4).

### Adaptive dynamics

#### Analysis

The analysis above considers competition between producers and completely non-producing LOF mutants, a much coarser comparison than would arise through incremental evolutionary change. In practice, non-producing strains may arise through gradual reductions in production rates as opposed to spontaneous loss of function. We use adaptive dynamics (Box 2) to determine the evolutionary stability of public goods production.

**Box 2: Overview of adaptive dynamics**

Adaptive dynamics [63–65] is a standard analytical approach that views evolution as a series of invasion attempts by new mutants into a resident population at equilibrium [64, 66, 67]. The *invasion growth rate* (*G*; the growth rate of a vanishingly rare mutant in the environment dominated by the resident) is the key quantity of analysis. A successful mutant has a positive invasion growth rate, thus increasing in frequency and causing the population to evolve toward the mutant trait. If the invasion growth rate is negative, the invader goes extinct and no evolution occurs. The derivative of the invasion growth rate with respect to the trait in question, *the fitness gradient*, determines the direction of evolution: selection favors higher trait values at positive fitness gradients and lower trait values at negative fitness gradients. The goal of adaptive dynamics is to find uninvasible strategies, known as evolutionarily stable strategies (ESS). An ESS is, as a resident, resistant to invasion from any other trait value and thus is an evolutionary equilibrium.

**Fig 5:**
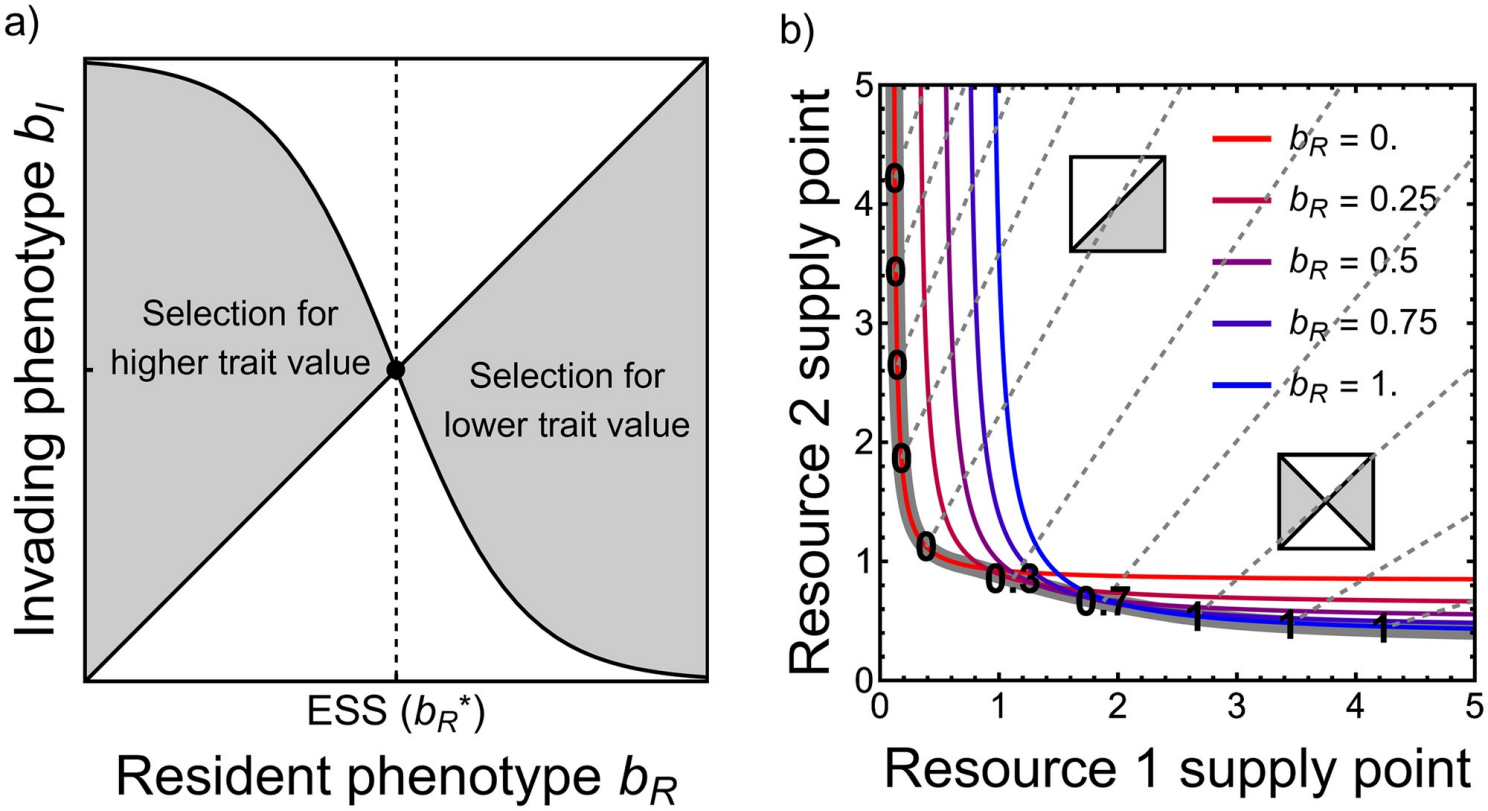
Overview of adaptive dynamics (see Box 2 for details). (a) Example pairwise invasibility plot (PIP). Shaded regions indicate pairs of resident (x-axis) and mutant (y-axis) trait values that result in a positive invasion growth rate for the mutant and thus predict a successful invasion; white regions indicate a negative invasion growth rate and loss of the mutation. An ESS is any resident trait value at which a vertical line passes through only white regions. (b) ZNGIs for different resident trait values (colors) as a function of the resource environment. The thick gray line marks the geometrical envelope, defined by the ESS strategy at any supply point (i.e., following the outermost ZNGI). Numbers on the envelope list select ESS values along this envelope and the dashed lines are their impact vectors (see Box 1).

Adaptive dynamics can be visualized using pairwise invasibility plots (PIPs; Fig 5a). PIPs plot resident trait values, *b*_*R*_, against invading trait values, *b*_*I*_, and shade trait combinations for which the invader has a positive invasion growth rate gray. Along the main diagonal, invader and resident are identical, so that the invasion growth rate must be 0 (i.e., equal to the resident’s net growth rate at its equilibrium). The location of successful invasion above or below this diagonal line reveals the direction of evolution. Thus, if gray (successful invasion) is above the diagonal and white is below, then the population evolves toward higher trait values, and vice versa. Because ESS residents are by definition uninvasible, the graphical signature of an ESS is that a vertical line passes through no gray regions (e.g., dashed line in Fig 5a below).

In single-resource systems, the ESS is the strategy that minimizes R* [34, 68]. The situation is more complex with two resources. There, a strategy is an ESS if its resource levels at equilibrium (represented by a point in the resource plane; Fig 5b) are located below the zero net growth isoclines (ZNGIs; Box 1) of all other strategies, ensuring that no other strategy can invade. This means that all the possible ESS can be found by plotting the ZNGIs of a dense collection of strategies (colored lines in Fig 5b, illustrative sample) and keeping only the outermost points from that collection (thick gray line) which gives their *geometrical envelope* [62]. Thus, each point on the envelope is an ESS with phenotype (numbers displayed on the envelope, illustrative sample) matching the strategy from whose ZNGI that point stems. Then, similar to the ecological version of the approach (Box 1), one can “follow” the impact vectors (dashed gray lines) to map a given supply point its the corresponding ESS (e.g. inset PIPs along two of the impact vectors). This graphical approach summarizes the evolutionary outcome of both trait values and resource levels obtained along varying supplies of the two resources, making it possible to visualize how the environmental context influences evolution.

#### Results

First, consider the evolution of siderophore production rate *q*_*i*_. Production of the fully public siderophores we consider is never evolutionarily stable (*q*_*i*_ always evolves to 0; S4 Appendix). Since siderophores cannot be privatized in our model, this agrees with the intuition from our single-resource model and further illustrates that siderophores’ role in partially privatized N-fixation cannot lead to an evolutionary benefit of siderophore production, though we saw above that siderophore producers may ecologically outcompete siderophore non-producers (e.g., Fig 4civ).

**Fig 6:**
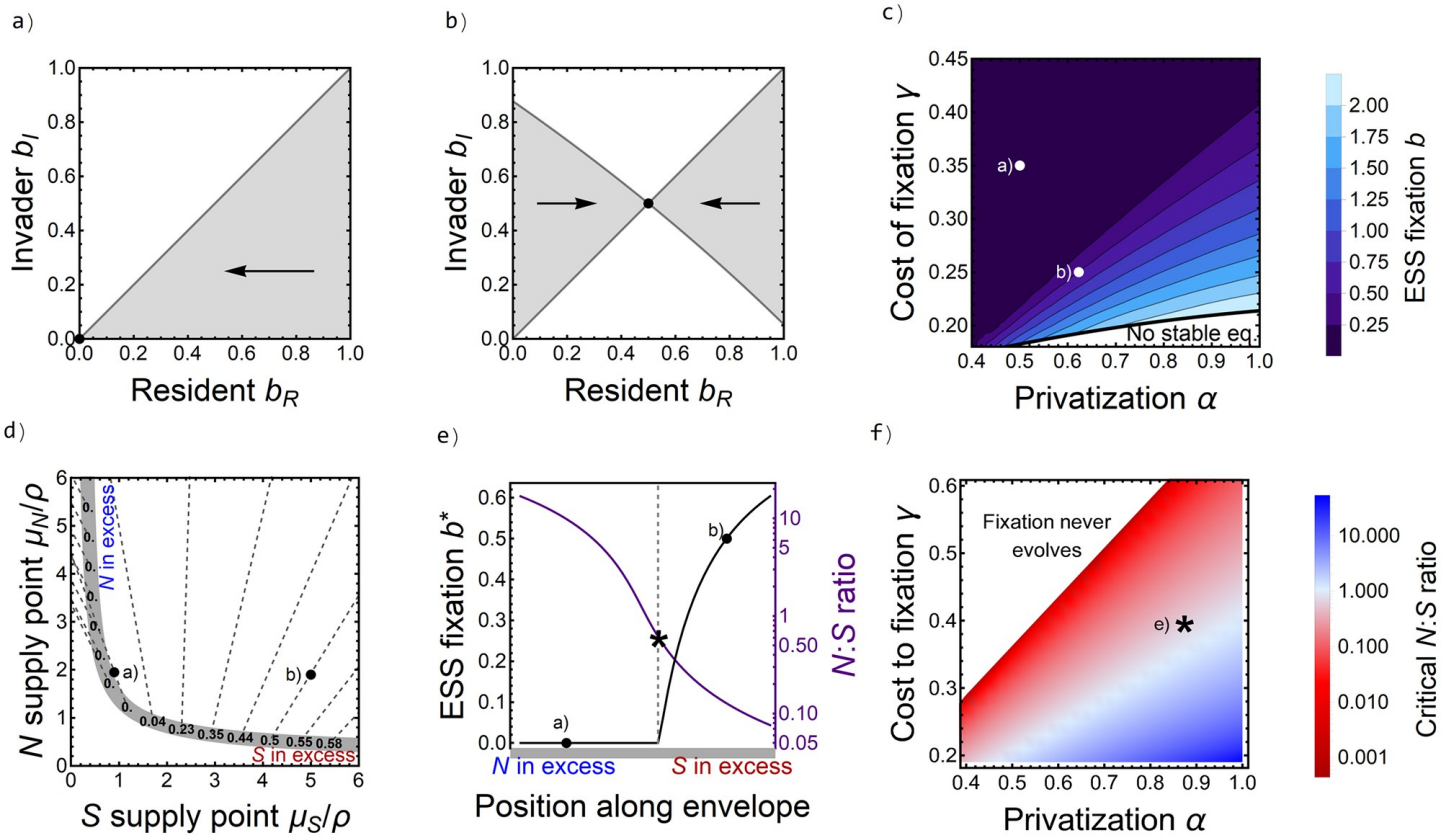
Adaptive dynamics in the colimitation model. (a) Pairwise-invasibility plot (PIP; see Box 2) using *α* = 0.5 and *γ* = 0.35 (marked by dots in panels c, d, and e) with gray indicating regions of successful invasion and arrows indicating the direction of evolution. The ESS, which here represents loss of fixation, is marked with a dot. (b) PIP using *α* = 0.62 and *γ* = 0.25 (marked by dots in panels c, d, and e) showing that evolution leads to an intermediate ESS maximum fixation rate. (c) ESS fixation *b* as a function of privatization *α* (horizontal axis) and cost of fixation *γ* (vertical axis) with lighter colors corresponding to higher ESS *b*. Note that low fixation costs result in unbounded growth (hence the “no stable equilibrium” region). (d) Geometrical envelope (see Box 2) shown by thick gray line, with ESS *b* values (black numerals) and their corresponding impact vectors (dashed lines) shown for points along the envelope. PIPs qualitatively the same as a) and b) are mapped onto the impact vectors. (e) ESS fixation rate (black line, left axis) and *N*:*S* ratio at equilibrium (purple line, right axis) plotted as functions of position along the geometrical envelope from d), moving from the siderophore-limited to N-limited regimes. To the left of the dashed line (i.e. for *N*:*S* ratios greater than the starred value), there is no fixation at equilibrium, as in a); for lower *N*:*S* ratios, there is an intermediate ESS fixation rate as in b). (f) Critical *N*:*S* ratio where fixation transitions from 0 to positive, marked by star on e). Parameters as shown in Table 1.

We see two qualitatively distinct outcomes for the evolution of N fixation, visible in the pairwise invasibility plots (PIPs; Box 2). In the first regime, mutants with lower fixation rates can always invade residents with higher fixation rates and, as a result, fixation is gradually lost (Fig 6a). In the second regime, there is a single non-zero evolutionarily stable strategy (ESS) toward which the fixation rate will evolve (Fig 6b). The transition between these two regimes is shaped by environmental conditions and occurs as the system switches from being siderophore-limited (when not fixing is favored) to N-limited (when fixing is favored) (Fig 6d). By examining the critical *N*:*S* ratio at which the transition between these two regimes occurs (Fig 6e, asterisk), we find that when costs are sufficiently low and privatization sufficiently high, more siderophore-limited systems have an ESS of non-zero fixation (Fig 6f). In contrast, when costs are high and privatization low, systems must become more N-limited before fixation is favored (Fig 6f). By keeping supply points constant, we see that decreasing fixation costs and increasing privatization not only determine the transition between the two regimes, but generally also lead to increasing ESS fixation rates (Fig 6c).

The fact that fixation reaches an intermediate ESS in the colimitation model, rather than the runaway evolution of even higher fixation rates (as seen in the single-resource model), is a consequence of colimitation. Cells must balance their ability to grow on both resources: in contrast to the single-resource model, here there is no benefit to becoming better at total nitrogen aquisition *ad infinitum* because growth is a saturating function of N acquisition and thus cells cannot always increase their growth rate by acquiring more nitrogen.

## Discussion

Here, using a resource-explicit framework, we demonstrate the importance of the external environment for the production of partially privatized public goods. Both in ecological competition and in evolution, the rate at which resources enter the system critically alters the competitive ability of resource producers that are most competitive when the resource is limiting in the environment. Our results show that the role privatization plays in stabilizing public goods production in previous phenomenological models holds in mechanistic consumer-resource models. Thus, microbial communities in the relatively unstructured and mixed environments in the open ocean that stably produce public consumables [18, 19] can only do so because the “public goods” are, in fact, not fully public. However, producers of fully public goods can outcompete non-producers if producers are also benefiting sufficiently from the production of a partially privatized good. By explicitly modeling the external environment, we show that the conditions leading to production being a competitive advantage are shaped by the environmental availability of the resource, with production of that resource being more favorable when the resource is limiting. We also demonstrate that the roles of privatization and the environmental context hold even in the realistic case that “producers” only facultatively produce the resource dependent on external concentrations. Differences between our two models highlight the role metabolic constraints such as colimitation play in eco-evolutionary dynamics. Finally, the rarity of coexistence in our model relative to past work demonstrates the importance of feedbacks between cell populations and the external environment in shaping competitive outcomes. In sum, producers are most likely to win when the produced resource is highly privatized, not very costly to produce, and limiting to population growth.

### Privatization: Evolution, implications, and limitations

Given the clear benefits to privatization, why do resources remain leaky rather than becoming fully privatized? Selection always favors a reduction in leakiness (increasing *α*) in our model (S4 Appendix). Thus, at least in systems with weak spatial structures like the open ocean, leakiness is likely not an adaptation but rather a physical constraint, especially for more diffusible molecules like ammonia. Loss to the environment could in principle be reduced (i.e., privatization could be increased) by membrane adaptations, but it may be less costly to simply fix more nitrogen than to meet the energetic demands of such defenses against leakiness. Similarly, privatizing siderophores (e.g., by making chemical modifications that increase their affinity with the producer’s outer membrane) can be costly because they are more efficient at acquiring iron when they have high diffusivity [50]. Despite such bioenergetic constraints, privatization can arise as an adaptation when microbes are faced with cheaters against which they must defend the consumables they produce [69, 70].

For microbial communities, resource defensibility depends most fundamentally on encapsulation by the cell membrane, a fundamental spatial structuring that persists even when mixing prevents the positive assortment of cooperators. Without the boundary the membrane creates, there can be no private benefits. The extent to which the membrane enables privatization for a given good depends on both the chemical nature of the good and cellular location of production relative to the membrane. Large storage molecules like polyhydroxybutyrate and polyphosphates (for carbon and phosphate storage, respectively), and enzymes that require active transport in or out of the cell, are effectively made fully private by the membrane. By contrast, the membrane presents a relatively low barrier to exit for small, diffusible compounds (e.g., ammonia). When such goods are produced by periplasmic or membrane-bound enzymes, they need only travel a short distance before leaking away from the producer as public goods, and privatization will tend to be low. Even for diffusible goods, privatization can be higher when production is localized to the cytoplasm and products are not actively secreted. In this case, a product molecule’s path length to the membrane will be longer, affording the producer a better chance of forestalling loss by capturing those goods for private use or vacuolar storage along the way.

### Cross-feeding mutualism and coexistence

Although we have focused primarily on competitive exclusion, coexistence can occur in our model under restricted conditions. Its rarity can largely be attributed to high amounts of niche overlap, since we consider competing strains that differ only in their resource production ability, with all other traits (parameter values) held equal [28, 71]. The rarity of coexistence observed here stands in contrast to past work in phenomenological models that predicts coexistence should be common on partially privatized goods due to negative frequency dependence. This difference with past work underscores the importance of considering the external environment. Both the abiotic conditions and the strains’ effect on the environment must be appropriate for coexistence to occur.

Coexistence between competing strains as well as the evolutionary stability of microbial resource production has been thoroughly considered for cross-feeding mutualisms (when two cell strains use one another’s metabolic byproducts for growth; [72, 73]). Again, cross-feeding mutualisms are often studied in the context of spatial structure [74–76]. Spatial structure can promote mutualistic dependence instead of the genomic “race to the bottom” described by the BQH [77], but public goods must be inherently leaky for these spatial effects to persist in the face of evolutionary changes [78]. Work carried out on well-mixed systems has shown that increasing resource supply from the environment can stabilize coexistence [79], but cross-feeding is known to allow for stable coexistence of mutualists even in the absence of external resource supply [80]. Privatization helps determine the result of these interactions, shaping the composition of the microbial community [22].

### Relationship to the Black Queen Hypothesis and social evolution theory

The BQH paradigm is prevalent in the current understanding of the stable production of public goods in well-mixed microbial communities [81]. From the perspective of the BQH [31, 32], producers are viewed as losers in a “race to the bottom” [29, 30]. Our results suggest a shift in perspective. Rather than being losers, producers are strains whose optimal strategy under a given set of environmental conditions is to produce the resource. There is no special selective force stopping the only strain producing a resource from gradually losing the ability to produce the resource and thus driving the system to collapse (see also [80]). As such, evolutionary suicide (where a species drives itself extinct through adaptive evolution; equivalently, the tragedy of the commons; [8]) is a nontrivial possibility [82, 83] and one that would benefit from greater consideration in the context of microbial public goods production.

The BQH suggests that evolutionary suicide is prevented and producer/beneficiary coexistence promoted by negative frequency dependent selection, where each strain’s fitness is higher when it is rare than when it is common [32]. Whereas a formal model of public services like detoxification [22] often predicts coexistence, in part because death (their Eq 2) is explicitly modeled as strongly negatively frequency dependent, our model of public consumables predicts coexistence only rarely. In our model, which strain has highest fitness does not always depend on frequency, for two reasons. First, producing a resource may be too costly and insufficiently private to provide an advantage regardless of environmental conditions. Second, environmental conditions (i.e., external resource supplies) may be such that resource production is always favorable or always unfavorable, regardless of the makeup of the community. In other words, even with negative frequency dependence, the fitness curves of a producer and LOF mutant may not cross. Future mechanistic modeling would help bridge our results and those of [22]; at present, it is unclear whether differences in our conclusions are due to fundamental differences between the two classes of public goods (services vs. consumables), between obligate and facultative resource production, between the underlying model structure (explicit modeling of the public good vs. implicit accounting via frequency dependence), or a combination of these. More broadly, the potential for the production of leaky public goods to diverge from BQH assumptions demonstrates the importance of mechanistic modeling and a consideration of resource production that may be facultatively turned on or off based on the environment.

Social evolution theory provides a broad perspective on public goods production as a type of cooperation. Since resource production gives an individual a direct fitness benefit in our model, partially privatized microbial public goods production is an example of the “direct benefits” path to the evolution of cooperation [84, 85]. That is, public goods production only evolves in well-mixed systems when conditions are such that resource production is a net benefit to population growth. Our results (in particular inequalities (15) and (17)) provide a striking example of the importance of the environmental context for social evolution [86] as well as the potential for feedbacks between social trait evolution and environmental conditions [27, 87]. Viewed in this light, this study connects the BQH, social evolution, and resource-explicit community ecology [26]. In the language of social evolution theory, the stability of investment into public goods production is challenged by “cheaters” that reap the benefits of resource production without paying production costs [88]. Phylogenetic analyses have shown that cheating is common for the production of public iron-acquisition goods like siderophores [18, 89] and thus a significant problem for sustained resource production in microbes. Partial privatization and spatial structure are thus features of systems that limit the effectiveness of cheating and prevent over-exploitation by cheaters [3, 90–92].

### Conclusions

We have shown that public resource production is likely to persist when 1) private benefits are large (the resource is not very leaky), 2) the costs of production are low, and 3) environmental conditions make population growth highly sensitive to increased resource acquisition. We derive an explicit lower bound (inequalities (15) and (17)) on the level of privatization, answering the question: “how public can public goods be?” We confirm that the central role of privatization found previously [20, 22] holds in a resource-explicit framework, but find that how public leaky goods can be depends critically on the environmental context (i.e., external resource concentrations). Our results also make clear that trade-offs in competitive abilities for the resources will not necessarily lead to coexistence between producers and cheaters in a resource-explicit framework. In contrast to the perspective that producers have lost in the “race to the bottom”, our results demonstrate that privatization leads to a “selfish” continuation of resource production.

## Supporting information

S1 Table Coexistence in the colimitation model. (PDF)

S1 Fig Additional cases from single resource model. (PDF)

S2 Fig Parameter effects on strain viability. (PDF)

S3 Fig Parameter effects on strain invasibility. (PDF)

S4 Fig ZNGIs for three pairwise competition cases. (PDF)

S5 Fig Two coexistence equilibria in the LOFN vs LOFS case. (PDF)

S6 Fig Parameter effects on N*. (PDF)

S7 Fig Position of Full’s impact vectors. (PDF)

S1 Appendix Formal analysis of the single-resource model. (PDF)

S2 Appendix Analytical results in the limit of one resource in excess. (PDF)

S3 Appendix The relative ordering of impact vectors. (PDF)

S4 Appendix Adaptive dynamics in the colimitation model. (PDF)
