# Supplementary material for: How public can public goods be? Environmental context shapes the evolutionary ecology of partially private goods
Source: PLoS Comput Biol. 2022 Nov 1;18(11):e1010666. doi: 10.1371/journal.pcbi.1010666 (PMC9651594; doi:10.1371/journal.pcbi.1010666)
Supplement: S1 Table — (PDF) [file pcbi.1010666.s001.pdf]

S1 Table: Coexistence in the colimitation model

Table S1. Percentage of parameter combinations for which both strains are viable that result in either stable coexistence or a priority effect for each of the six pairwise competition scenarios. Abbreviations in Fig. 2.

	**Stable coexistence**	**Priority effect**
**Full vs LOFN**	0.7	0.0
**Full vs LOFS**	0.0	0.0
**Full vs LOFB**	0.6	0.6
**LOFN vs LOFS**	0.7	9.5
**LOFN vs LOFB**	0.0	0.0
**LOFS vs LOFB**	2.9	0.0
