# Supplementary material for: How public can public goods be? Environmental context shapes the evolutionary ecology of partially private goods
Source: PLoS Comput Biol. 2022 Nov 1;18(11):e1010666. doi: 10.1371/journal.pcbi.1010666 (PMC9651594; doi:10.1371/journal.pcbi.1010666)
Supplement: S1 Fig — (PDF) [file pcbi.1010666.s002.pdf]

S1 Figure: Additional cases from single resource model

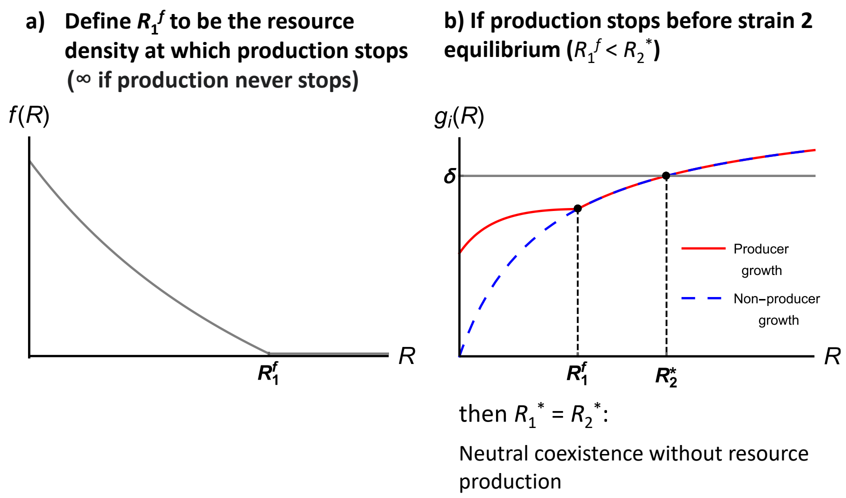

Fig S1. Overview of results from the single-resource model. (a) Graphical representation of minimum resource concentration without production, R_1^f, a key quantity for determining external mortality. (b) The strains' per capita growth rates excluding external mortality g_i as a function of resource density R. When production stops at lower resource densities than the non-producer's equilibrium ($R_1^f < R_2^*$), then the two strains neutrally coexist with no production at equilibrium. Because the two strains are functionally equivalent for $R > R_1^f$, $R_1^* = R_2^*$ in this case.
