# Supplementary material for: How public can public goods be? Environmental context shapes the evolutionary ecology of partially private goods
Source: PLoS Comput Biol. 2022 Nov 1;18(11):e1010666. doi: 10.1371/journal.pcbi.1010666 (PMC9651594; doi:10.1371/journal.pcbi.1010666)
Supplement: S2 Fig — (PDF) [file pcbi.1010666.s003.pdf]

S2 Figure: Parameter effects on strain viability

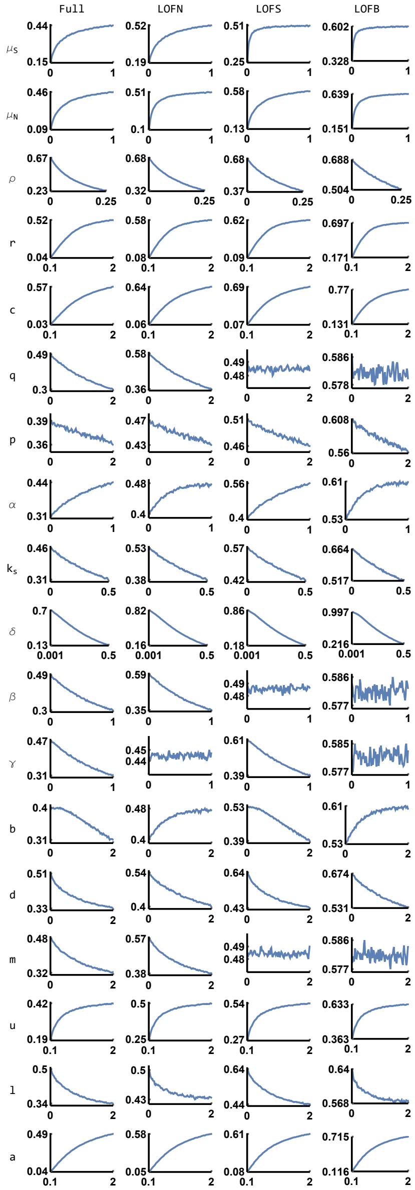

Fig S2. The probability that each resident is viable as each parameter changes across its range. Note that the y-axis scale changes for each plot. See Table 1 for the meaning of each parameter.
