# Supplementary material for: How public can public goods be? Environmental context shapes the evolutionary ecology of partially private goods
Source: PLoS Comput Biol. 2022 Nov 1;18(11):e1010666. doi: 10.1371/journal.pcbi.1010666 (PMC9651594; doi:10.1371/journal.pcbi.1010666)
Supplement: S3 Fig — (PDF) [file pcbi.1010666.s004.pdf]

S3 Figure: Parameter effects on strain invasibility

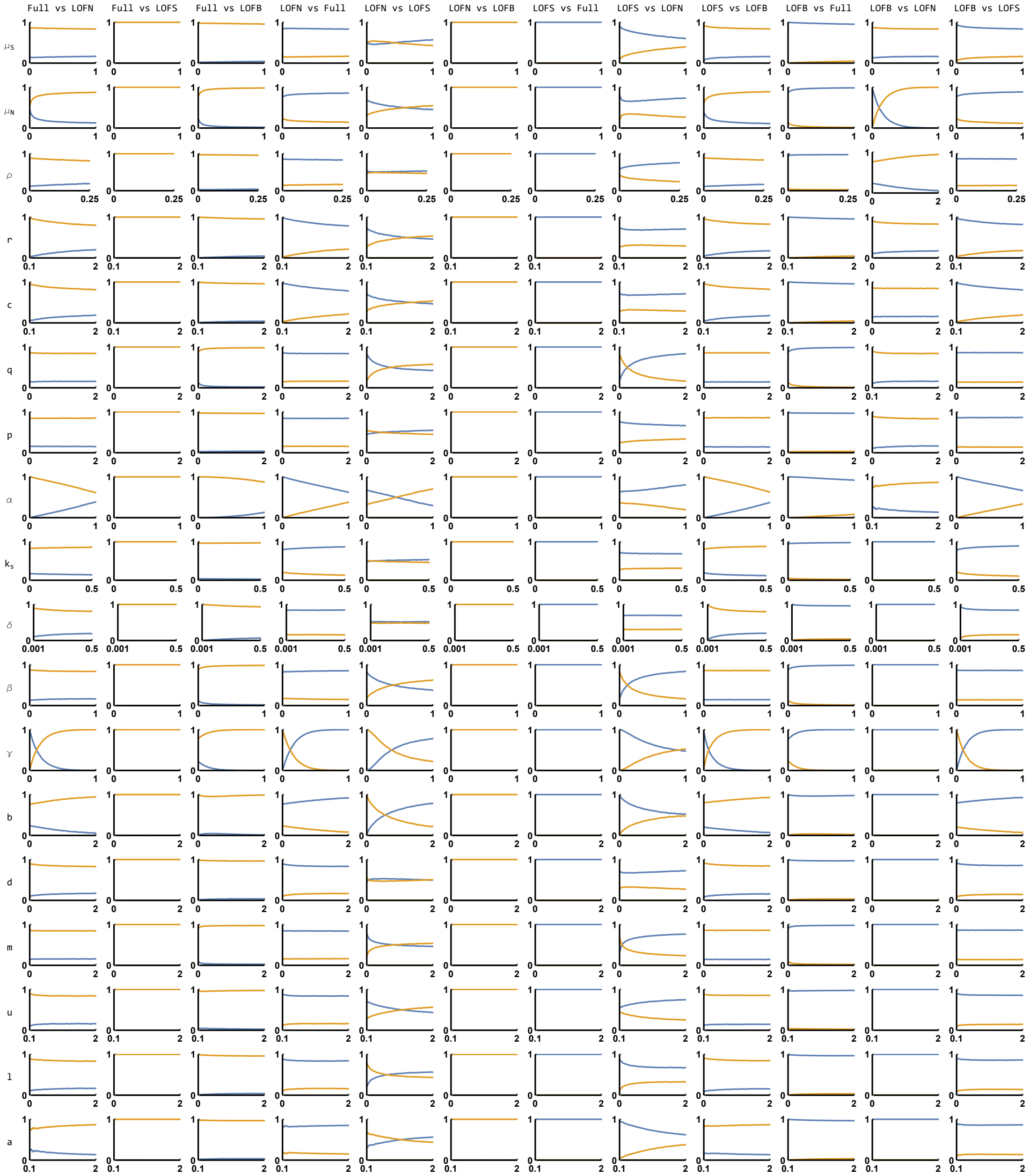

Fig S3. The probability that each resident (listed first) is able to resist invasion by each invader (listed second). Blue is the probability the invasion is unsuccessful (resident persists) and orange is the probability that the invasion is successful. See Table 1 for the meaning of each parameter.
