# Supplementary material for: How public can public goods be? Environmental context shapes the evolutionary ecology of partially private goods
Source: PLoS Comput Biol. 2022 Nov 1;18(11):e1010666. doi: 10.1371/journal.pcbi.1010666 (PMC9651594; doi:10.1371/journal.pcbi.1010666)
Supplement: S4 Fig — (PDF) [file pcbi.1010666.s005.pdf]

S4 Figure: ZNGIs for three pairwise competition cases

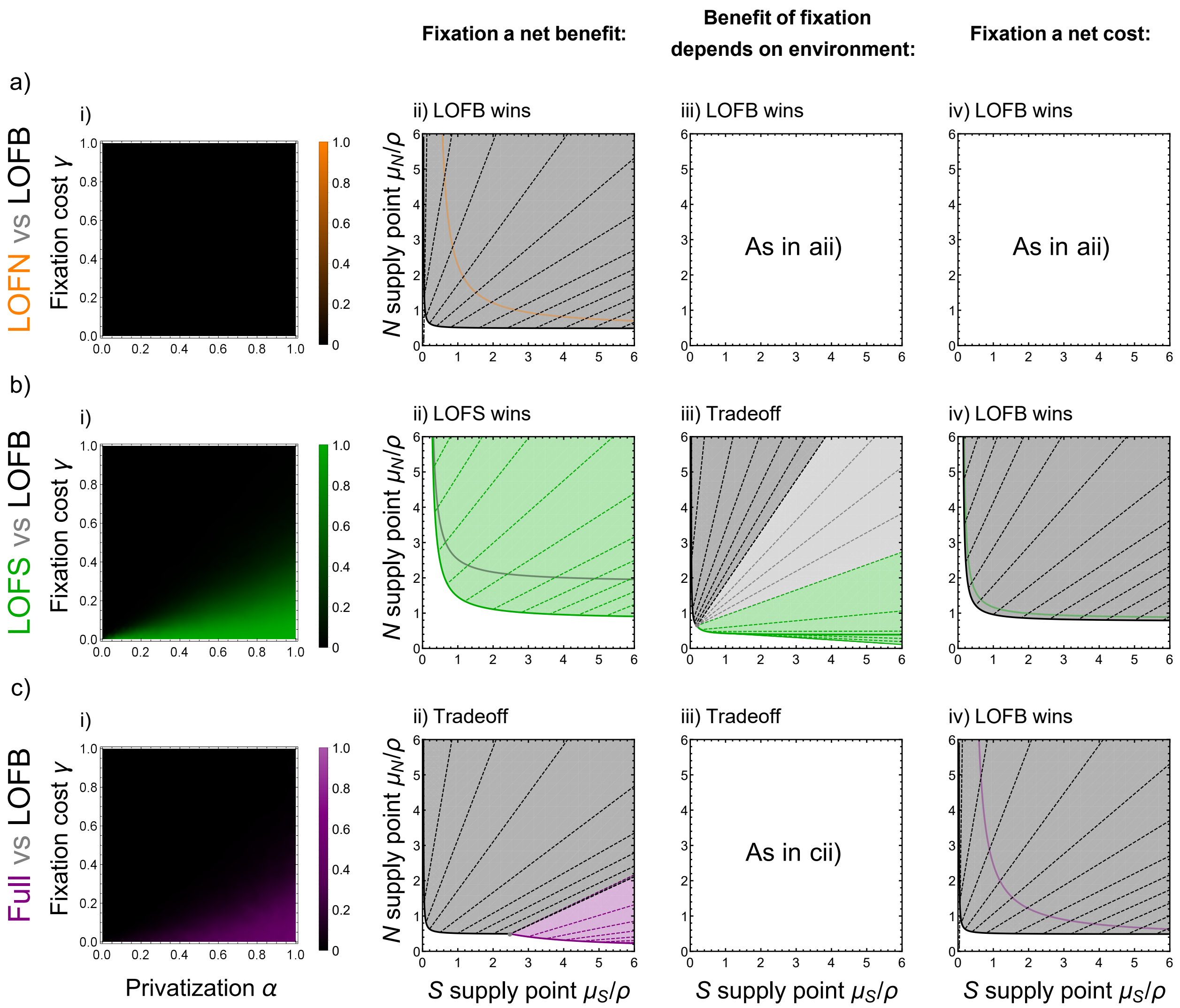

Fig S4

Fig S4. ZNGIs and sensitivity analysis for the role of privatization α and cost to fixation γ for the three pairwise competition scenarios not shown in main text Figure 4 (rows). The first column of each row (i) shows the probability that the invasion was successful for the second strain listed. The second column of each row plots the ZNGIs and impact vectors (see Box 1) when fixation is beneficial even when siderophores are limiting ($\mathscr{S}(S^*, \infty) > \gamma/\alpha$). The third column of each row plots the ZNGIs and impact vectors when fixation is beneficial when nitrogen is limiting but costly when siderophores are limiting ($\mathscr{S}(\infty, N^*) > \gamma/\alpha > \mathscr{S}(S^*, \infty)$). The final column in each row plots the ZNGIs and impact vectors when fixation is costly even when nitrogen is limiting ($\gamma/\alpha > \mathscr{S}(\infty, N^*)$). (a) LOFN vs. LOFB. i) Regardless of parameters, LOFB can successfully invade the LOFN's resident equilibrium ii-iv) because the ZNGI for LOFB always falls below the ZNGI for LOFN. (b) LOFS vs LOFB. i) LOFS can resist invasion from LOFB if fixation is private (high α) and not very costly to produce (low γ). ii) If fixation is always a net benefit, then LOFS always wins. iii) If the benefit of fixation depends upon the environment, then either strain could win or they could coexist (with the outcome depending on environmental conditions). iv) If fixation is always a net cost, then LOFB always wins. (c) Full vs. LOFB. i) Full can resist invasion by LOFB if fixation is private (high α) and not costly to produce (low γ). ii-iii) If fixation is a benefit when nitrogen is limiting, then there is a trade-off with the winner determined by environmental conditions. iv) If fixation is costly when nitrogen is limiting, then LOFB always wins.
