# Supplementary material for: How public can public goods be? Environmental context shapes the evolutionary ecology of partially private goods
Source: PLoS Comput Biol. 2022 Nov 1;18(11):e1010666. doi: 10.1371/journal.pcbi.1010666 (PMC9651594; doi:10.1371/journal.pcbi.1010666)
Supplement: S5 Fig — (PDF) [file pcbi.1010666.s006.pdf]

S5 Figure: Two coexistence equilibria in the LOFN vs LOFS case

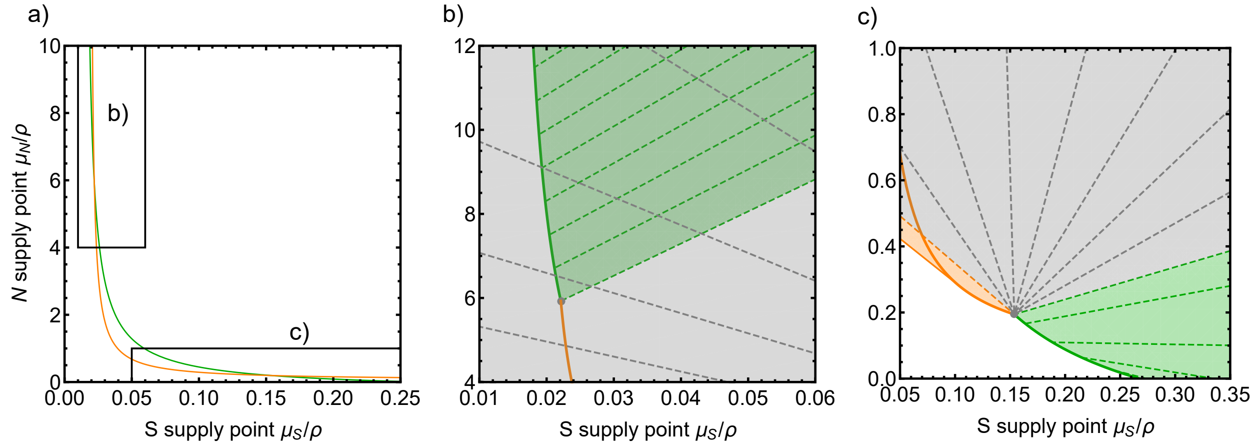

Fig S5. Two coexistence equilibria between LOFN and LOFS as a special case of when the benefit of fixation depends upon the environment. (a) ZNGIs cross at two points: once when *S* is limiting and once when *N* is limiting. (b) Coexistence equilibrium when *S* is limiting. As shown in S2 Appendix, at S^* LOFS always outcompetes LOFN. In this case, however, fixation is *more* costly moving away from S^* than is siderophore production (so LOFN begins to outcompete LOFS) and the ZNGIs cross. Tracing impact vectors, we see that all points trace to a stable coexistence equilibrium (shown in panel c). Some supply points may also map to alternative stable states with both strains extinct (outside of the ZNGIs) or LOFS excluding LOFN (green). The coexistence equilibrium shown in panel b is unstable. (c) Coexistence equilibrium when *N* is limiting. In this case, fixation is beneficial in the limit of *S* in excess and thus the ZNGIs cross again and LOFS outcompetes LOFN, producing stable coexistence. Note that some supply points lead to LOFS winning or LOFN winning (including an alternative stable state with both strains extinct; to the left of the orange ZNGI). The fact that stable coexistence is rare between LOFS and LOFN compared to a priority effect (S1 Table) suggests that the benefit of fixation depends on the environment case more often resembles Fig. 4ciii).
