# Supplementary material for: How public can public goods be? Environmental context shapes the evolutionary ecology of partially private goods
Source: PLoS Comput Biol. 2022 Nov 1;18(11):e1010666. doi: 10.1371/journal.pcbi.1010666 (PMC9651594; doi:10.1371/journal.pcbi.1010666)
Supplement: S6 Fig — (PDF) [file pcbi.1010666.s007.pdf]

S6 Figure: Parameter effects on N^*

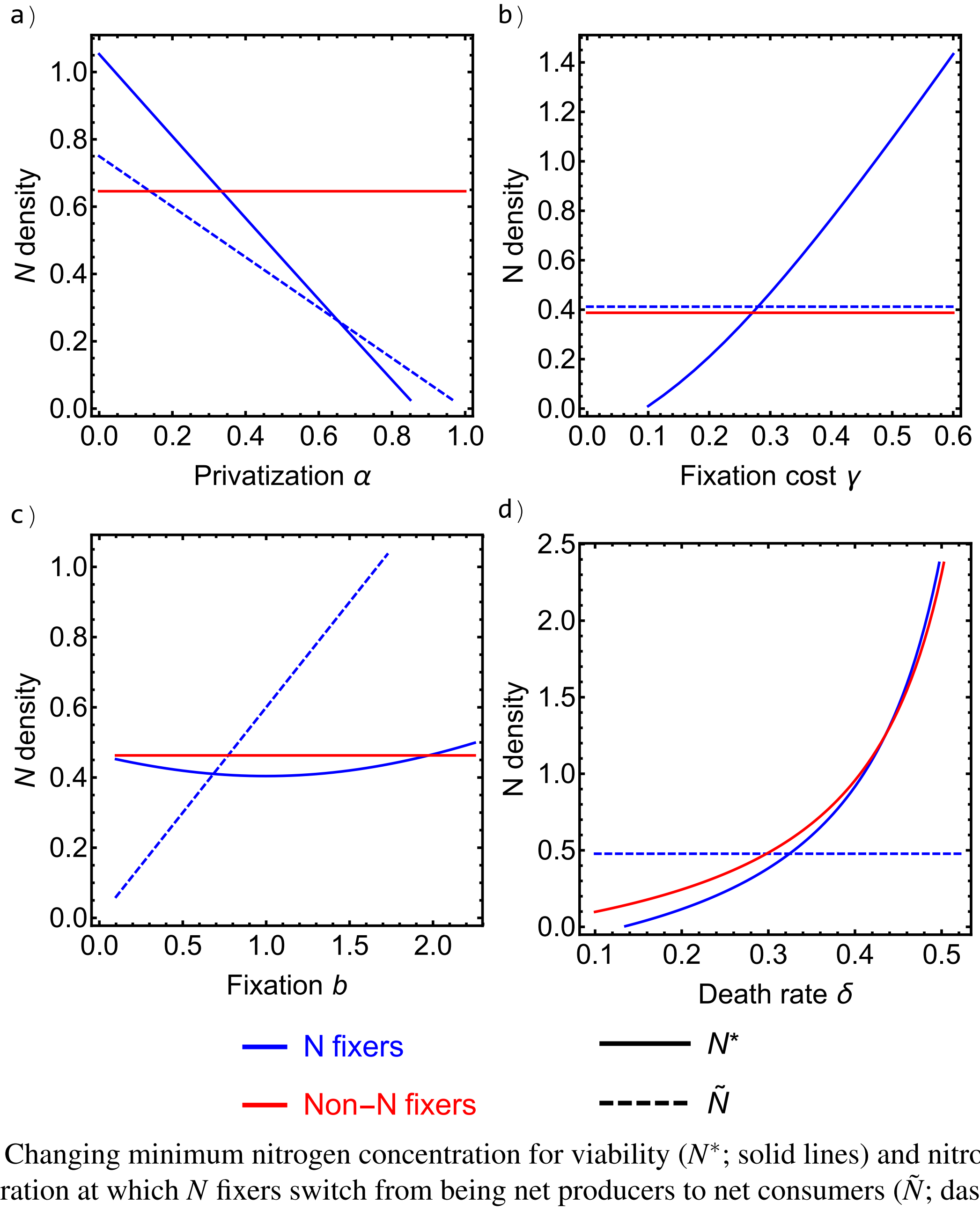

Fig S6. Changing minimum nitrogen concentration for viability (N^*; solid lines) and nitrogen concentration at which N fixers switch from being net producers to net consumers ($\tilde{N}$; dashed lines) as a function of (a) privatization α, (b) fixation cost γ, (c) maximum fixation rate b, and (d) mortality rate δ. N fixers correspond to blue lines and LOF mutants to red lines.
