# Supplementary material for: How public can public goods be? Environmental context shapes the evolutionary ecology of partially private goods
Source: PLoS Comput Biol. 2022 Nov 1;18(11):e1010666. doi: 10.1371/journal.pcbi.1010666 (PMC9651594; doi:10.1371/journal.pcbi.1010666)
Supplement: S7 Fig — (PDF) [file pcbi.1010666.s008.pdf]

S7 Figure: Position of Full's impact vectors

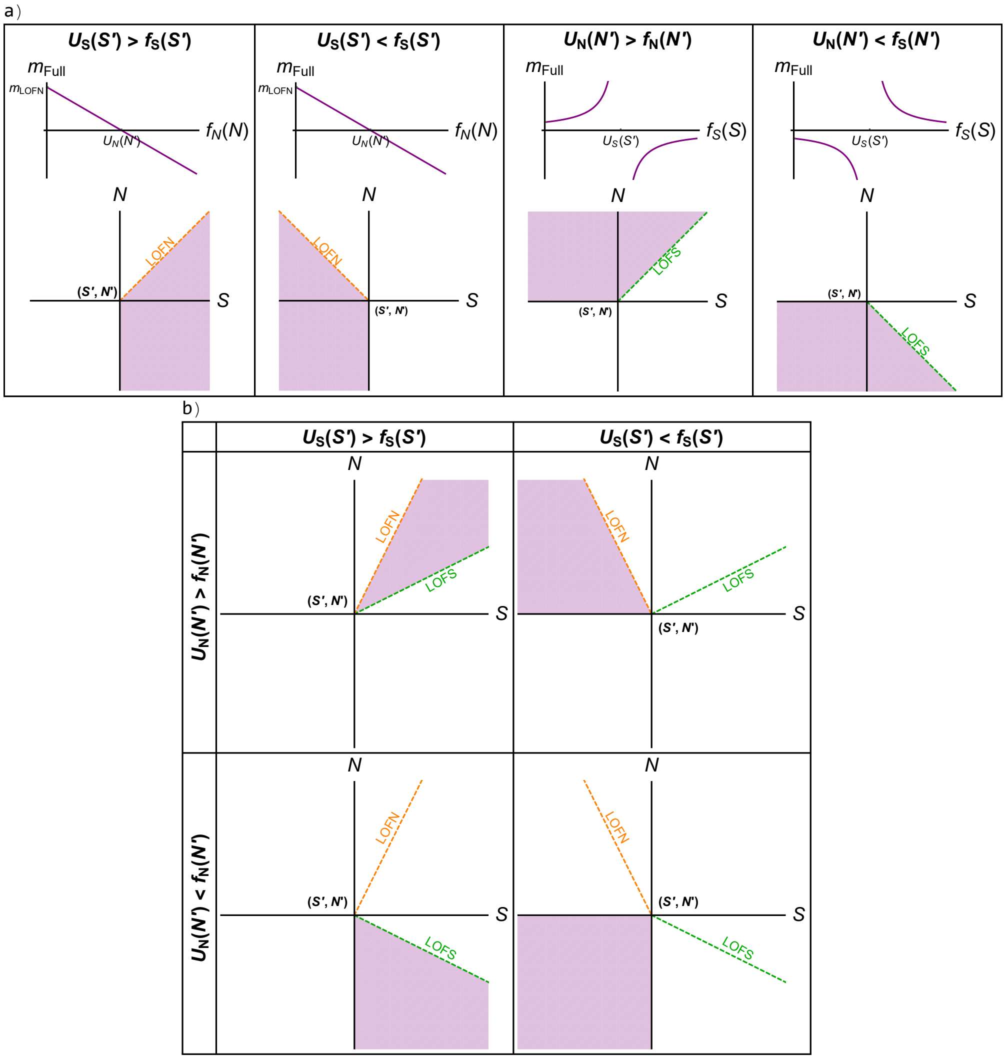

Fig S7. The relative position of the fully-functional strain's impact vector. Recall that the $S-N$ plane is translated such that (S', N') is the origin. (a) Top figure in each column shows the effect of resource production on m_{Full} in comparison with either m_{LOFS} or m_{LOFN}. The bottom figure then shows the way that the sign of $U_R - f_R$ constrains the possible positions of the fully-functional strain's impact vectors. Possible positions of its impact vector are shown in purple. (b) combines the panels from (a) to provide a complete set of constraints on Full's impact vector (in comparison to LOFN and LOFS) based on whether Full is a net producer or a net consumer of each resource at equilibrium. Possible positions of its impact vector are shown in purple.
