# Supplementary material for: How public can public goods be? Environmental context shapes the evolutionary ecology of partially private goods
Source: PLoS Comput Biol. 2022 Nov 1;18(11):e1010666. doi: 10.1371/journal.pcbi.1010666 (PMC9651594; doi:10.1371/journal.pcbi.1010666)
Supplement: S1 Appendix — (PDF) [file pcbi.1010666.s009.pdf]

S1 Appendix: Formal analysis of the single resource model

Brian A. Lerch, Derek A. Smith, Thomas Koffel, Sarah C. Bagby, Karen C. Abbott

Production halts before non-producers equilibrium

Since the production function $f(R)$ is constrained to be a non-negative, non-increasing function of resource density R, if production halts at a given resource density, then it also will not occur at any higher resource densities. We define R_1^f as the resource concentration at which production halts (S1 Fig a; formally, $R_1^f = \min\{R | f(R) = 0\}$). We will see below that R_1^f has important influence on competitive outcomes.

If the producer halts resource production at lower resource densities than the non-producer's resource equilibrium ($R_1^f < R_2^*$), then the two strains are ecologically equivalent at equilibrium ($R_1^* = R_2^*$; S1 Fig b) and no resources are produced at equilibrium. Coexistence of the two strains is only neutrally stable, following from the competitive exclusion principle that states that two species cannot stably coexist on one limiting factor (e.g. resource; [1, 2, 3]).

Adaptive dynamics

We use adaptive dynamics to study evolution in the single resource model (see Box 2 for an overview). We consider the evolution of a trait b that controls resource production f. Formally, we make this dependency explicit by writing $f(R,b)$. We assume that higher values of b correspond to higher values of f (i.e., $\frac{\partial f}{\partial b} > 0$). In adaptive dynamics, evolution is driven by the growth (at per capita rate G) of a mutant that is initially exceedingly rare, in the environment determined by the resident strain's equilibrium (Box 2).

Denoting the trait values of the resident and invading mutant as b_R and b_I, respectively, and $R_R^* = R^*(b_R)$ the resource level set by the resident at equilibrium, the invasion growth rate of strategy b_I in a system dominated by b_R is given by

$$G(b_I, b_R) = (\alpha - \gamma) f(R_R^*, b_I) - U(R_R^*) - \delta \tag{S1.1}$$

(i.e., the per capita growth rate from Eqs (1)-(2a) at resource level R_R^*). The direction of evolution is then given by the fitness gradient

$$\left.\frac{\partial G}{\partial b_I}\right|_{b_I=b_R} = (\alpha - \gamma) \left.\frac{\partial f(R_R^*, b_I)}{\partial b_I}\right|_{b_I=b_R}. \tag{S1.2}$$

Since $\frac{\partial f}{\partial b_I} > 0$ (increasing b increases production), this implies that the fitness gradient is positive (regardless of resident trait value) if and only if $\alpha > \gamma$. Thus, greater resource production will only evolve if the private benefits of producing a resource are greater than the production cost. This intuitive result confirms that privatization can confer an evolutionary advantage to public goods production in well-mixed systems under the assumptions of adaptive dynamics.

The remainder of our study aims to understand what these simple results imply for real systems. Does our conclusion that privatization can lead to the evolutionary stability of costly public good production in well-mixed systems when privatization outweighs costs ($\alpha > \gamma$) hold under more realistic scenarios such as colimitation by multiple resources?

Stability of the equilibrium between a single species and a single resource

Before looking at competition between a producer and a non-producer in the single resource model as presented in the main text, let us first examine the equilibrium properties of a generic species interacting with (i.e. consuming and potentially producing) a single resource. In monoculture, both the producer and the LOF mutant follow the general model

$$\frac{dx}{dt} = G(R)x \tag{S1.3}$$

$$\frac{dR}{dt} = \rho(\tilde{R} - R) + I(R)x. \tag{S1.4}$$

Where we use the notation $x = x_i$ for simplicity, $G(R) = g_i(R) - \delta$, $\tilde{R} = \mu/\rho$, and either $I(R) = (1-\alpha)f(R) - U(R)$ for the producer (if $i = 1$), or $I(R) = -U(R)$ for the LOF mutant (if $i = 2$). We will denote equilibrium cell densities and resource concentrations as $\hat{x}$ and $\hat{R}$, respectively. In this general model, the trivial equilibrium where the single consumer goes extinct occurs when $\hat{R} = \tilde{R}$ and $\hat{x} = 0$. Conversely, the non-trivial equilibrium where the consumer persists is given by $\hat{R} = R^*$ defined as satisfying the implicit equation $G(R^*) = 0$ and

$$\hat{x} = \frac{\rho(R^* - \tilde{R})}{I(R^*)}, \tag{S1.5}$$

which is feasible (i.e., biologically meaningful, $\hat{x} > 0$) when $R^* - \tilde{R}$ and $I(R^*)$ share the same sign. The Jacobian matrix associated with this system is

$$J(x,R) = \begin{pmatrix} G(R) & G'(R)x \\ I(R) & -\rho + I'(R)x \end{pmatrix} \tag{S1.6}$$

where primes indicate derivatives with respect to R. We now evaluate this Jacobian matrix at each of the two equilibria to evaluate their stability.

Consumer goes extinct: With $\hat{x} = 0$ and $\hat{R} = \tilde{R}$, we have

$$J(\hat{x},\hat{R}) = \begin{pmatrix} G(\tilde{R}) & 0 \\ I(\tilde{R}) & -\rho \end{pmatrix}. \tag{S1.7}$$

As the Jacobian matrix is triangular, its two eigenvalues are located on the diagonal; we conclude from the stability condition $G(\tilde{R}) < 0$ that the equilibrium where the consumer is extinct is locally stable if and only if the consumer cannot invade, i.e., its growth rate when rare is negative.

Consumer persists: When the consumer persists, the Jacobian J is

$$J(\hat{x}, \hat{R}) = \begin{pmatrix} 0 & G'(R^*)\hat{x} \\ I(R^*) & -\rho + I'(R^*)\hat{x} \end{pmatrix}. \tag{S1.8}$$

The eigenvalues of J, λ_- and λ_+, are related to the determinant and trace of this matrix:

$$\lambda_- \cdot \lambda_+ = \det J = -I(R^*) \cdot G'(R^*) \cdot \hat{x} \tag{S1.9}$$

$$\lambda_- + \lambda_+ = \operatorname{tr} J = -\rho + I'(R^*) \cdot \hat{x}. \tag{S1.10}$$

Under our assumptions, $I'(R)$ is always negative, so $\operatorname{tr} J$ is negative. Thus, by the Routh-Hurwitz stability criterion, a necessary and sufficient condition for stability is that $\det J > 0$. Because $\hat{x}$ has to be positive for this equilibrium to be biologically meaningful, the stability of the persistence equilibrium is controlled by the relative sign between the sensitivity of the consumer to the resource, $G'(R^*)$, and its impact on the resource, $I(R^*)$. In general, the impact and sensitivity need to have opposite signs for the persistence equilibrium to be stable, i.e., regulated by a negative feedback loop [4].

Considering the model in the main text, $G'(R)$ is always positive (population growth increases with more resource) for both the producer and the LOF mutant, so that stability is solely determined by the sign of $I(R^*)$. In the case of the LOF mutant, $I(R^*) < 0$ (it can only consume the resource), and the persistence equilibrium is always stable (as long as it is also feasible, which happens when $R^* < \tilde{R}$, i.e., when the resource supply is greater than the minimal amount of resource required for growth). Under these conditions, the extinction equilibrium is unstable, and persistence is thus a globally attracting equilibrium. A similar conclusion applies to the producer if $I(R^*) < 0$, i.e. if the producer is a net consumer around R^*.

However, it is also possible for the producer to be a net producer at R^*, i.e. $I(R^*) > 0$. There, even when this persistence equilibrium is feasible, which only happens when $R^* > \tilde{R}$ (the condition for $\hat{x} > 0$ in Eq (S1.5)), it will always be unstable. Under these conditions, the extinction equilibrium is stable. However, the presence of the unstable interior equilibrium signals the existence of another possible outcome: unbounded growth as a consequence of a positive feedback loop of the producer population on its resource. This points to an important, though intuitive, conclusion of the single resource model: the only way for a consumer to be properly regulated is if it is a net consumer of the limiting resource. If the effect of the consumer on that resource is net production instead, the resource cannot be limiting, regulation is impossible, and unbounded growth is practically unavoidable with the only other option being extinction, see [4].

Analysis of the two-strain model

With these general results in mind, let us look in more detail at the 4 possible equilibria of the single-resource model with the two strains presented in the main text. The possible equilibria are:

1) both consumers go extinct, 2) only the LOF mutant persists, 3) only the producer persists, or 4) coexistence.

1. Both consumers go extinct

This means that $\hat{x}_1 = \hat{x}_2 = 0$, and then we also get $\hat{R} = \tilde{R}$ from Eq (3) in the main text.

2. Only the LOF mutant population persists

This means that $\hat{x}_1 = 0$. Then, $\hat{R} = R_2^*$ from Eq (2b). Finally, Eq (3) gives:

$$\hat{x}_2 = \frac{\rho(\tilde{R} - R_2^*)}{\delta} \tag{S1.11}$$

hence this solution is feasible, i.e. positive, if and only if $\tilde{R} > R_2^*$.

3. Only the producer population persists

This means that $\hat{x}_2 = 0$. Then, we must have $\hat{R} = R_1^*$ from Eq (3), where R_1^* is the solution of the equation $(\alpha - \gamma)f(R_1^*) + U(R_1^*) - \delta = 0$. Under our assumptions, a positive R_1^* exists if and only if we have $(\alpha - \gamma)f(0) < \delta < (\alpha - \gamma)f(\infty) + U(\infty)$.

As a side note, because we always have $(\alpha - \gamma)f(0) < (\alpha - \gamma)f(\infty) + U(\infty)$ (because we assumed $g_1'(R) > 0$), there are only two ways the inequality above can be violated. 1) Resource production is too efficient, such that it completely overcomes R-limitation, which happens when $(\alpha - \gamma)f(0) > \delta$. For this to be true, it is necessary that $\alpha > \gamma$, i.e., that the producer reaps more direct benefits than costs for resource production. In this case, the producer population increases towards infinity. 2) Resource production is too costly overall, which happens when $\delta > (\alpha - \gamma)f(\infty) + U(\infty)$. For this to be true, as long as $U(\infty) > \delta$ (a requirement for non-producer viability), it is necessary that $\alpha < \gamma$, i.e. that the producers does not reap any direct benefits from resource production. It is also possible that $U(\infty) > \delta$ with $\alpha > \gamma$, but production benefits insufficient to overcome external mortality. In these cases, the producer population can only go extinct.

Let us assume that $(\alpha - \gamma)f(0) < \delta < (\alpha - \gamma)f(\infty) + U(\infty)$ so that that production is neither too beneficial nor too costly. Then again we have $\hat{R} = R_1^*$. From there, we get

$$\hat{x}_1 = \frac{\rho(\tilde{R} - R_1^*)}{\delta - (1 - \gamma)f(R_1^*)} \tag{S1.12}$$

where we have used the identity $\alpha f(R_1^*) + U(R_1^*) = \delta + \gamma f(R_1^*)$. As seen above, the impact $I(R_1^*) \equiv (1 - \gamma)f(R_1^*) - \delta$ plays a central role, as it captures the net effect of the producer on the resource in the neighborhood of its potential equilibrium in monoculture (see previous subsection). When $I(R_1^*) < 0$, the overall impact of the producer on the resource is consumptive and this equilibrium is stable when feasible. When $I(R_1^*) > 0$, the overall impact of the producer on the resource is net production, which initiates a positive feedback loop making this an unstable equilibrium.

First, note that $\gamma > 1$ implies that $I(R_1^*) < 0$; this means, however, that production always reduces the producer's growth rate compared to the LOF mutant regardless of the value of α. When $\gamma < 1$, there is the possibility for $I(R_1^*) > 0$, and its occurrence will depend on the production function f.

Then, if $I(R_1^*) > 0$ (e.g., producer has a net enriching effect on the resource) there are two possibilities. First, $\tilde{R} < R_1^*$ and the equilibrium point is positive but unstable (positive feedback loop), meaning that there are alternative stable states of i) the producer experiencing unbounded growth (R could either diverge or stabilize) and ii) the producer going extinct and the resource equilibrating at $\tilde{R}$. Second, $\tilde{R} > R_1^*$ and there is no positive equilibrium and the producer can only experience unbounded growth.

In the case that $I(R_1^*) < 0$ (the producer has a net consuming effect on the resource at R_1^*), then either 1) $\tilde{R} > R_1^*$ and the equilibrium point is positive and globally stable; or 2) $\tilde{R} < R_1^*$ and there is no positive equilibrium and the producer can only go extinct.

When the producer x_1 diverges to infinity, there are two possibilities for the behavior of R. First, note that the impact function $I(R) = (1-\alpha)f(R) - U(R)$ is strictly decreasing, starting at a positive value $I(0) = (1-\alpha)f(0)$ and ending at $I(\infty) = (1-\alpha)f(\infty) - U(\infty)$ (equal to $-\infty$ when $U(\infty)$ is infinite). We define R_1^c to be the value for which $I(R_1^c) = 0$, i.e. the resource level above which the net impact of the producer on the resource turns from productive to consumptive. If there is no such solution, let $R_1^c = \infty$.

With this quantity, the behavior of the system can be completely determined by the relative position between R_1^*, R_1^c and $\tilde{R}$. If $R_1^* < R_1^c$, the system possesses a divergent trajectory where $x_1 \to \infty$ and $R \to R_1^c$. If $\tilde{R} < R_1^*$, the trivial equilibrium where $\hat{x}_1 = 0$ and $\hat{R} = \tilde{R}$ is stable. Note that both conditions can happen at the same time, leading to alternative possible outcomes dependent on initial conditions as discussed above (one stable state and one divergent trajectory). Lastly, when both conditions above are false (i.e., $\tilde{R} > R_1^* > R_1^c$), there is a single, non-trivial, equilibrium and it is stable.

4. Coexistence

The producer and LOF mutant may both persist at equilibrium. This can happen in the special case where $\alpha = \gamma$; the two strains are then neutral in the sense that their growth rates are equal for all R, but only one of them produces the resource. Equilibrium coexistence may also occur if $f(R_2^*) = 0$; then there is no resource production at equilibrium and the two species are effectively neutral in the neighborhood of the coexistence, both behaving as LOF mutant consumers.

The producer and LOF mutant may also both persist if the producer is diverging to infinity and $R = R_1^c$. Then, if $R_1^c > R_2^*$, the LOF mutant can invade. Through numerical analysis we found evidence that both can persist on a trajectory to infinity with resource concentrations stabilizing at $\tilde{R}$.

Summary of results from Single Resource Model

Let us reorganize the results derived above around a step-by-step guide that presents how to analyze the single resource model and conclude on the outcome of the competitive dynamics.

First, compute the supply $\tilde{R}$, the value at which resources equilibrate in the absence of the producer and the LOF mutant.

Then, consider the LOF mutant. Compute R_2^*, the value of the resource for which mutant growth $g_2(R)$ is zero (formally $U^{-1}(\delta)$; this number could be infinite when the LOF mutant can't grow under any resource availability). In monoculture, the LOF mutant reaches a positive, stable state if and only if there is enough resource supply, i.e., $R_2^* < \tilde{R}$. If $R_2^* > \tilde{R}$, the consumer goes extinct.

Next, turn to the producer in monoculture. Compute R_1^*, the value of the resource for which producer growth $g_1(R)$ is zero (take R_1^* negative or infinite when there is no finite, positive solution). Next, compute R_1^c, the value of the resource for which the net impact of the producer on the resource is exactly zero, i.e., $(1-\alpha)f(R_1^c) = U(R_1^c)$ as production compensates uptake (again, if this doesn't happen, take R_1^c infinite). With these two quantities, the behavior of the producer in monoculture can be completely determined by the relative position between R_1^*, R_1^c and $\tilde{R}$. If $R_1^* < R_1^c$, then the producer would be a net producer at the resource level that would regulate it. This situation cannot be a stable equilibrium and leads instead to a divergent trajectory where $x_1 \to \infty$ and $R \to R_1^c$ (as a phase plane analysis demonstrates). Ecologically, this situation corresponds to the growth of the producer remaining positive with resource levels equilibrating so that the net effect of the producer on the resource is exactly zero. If $\tilde{R} < R_1^*$, then the trivial equilibrium where $\hat{x}_1 = 0$ and $\hat{R} = \tilde{R}$ is stable, as the producer goes extinct. Note that these conditions are not mutually exclusive, in which case either the extinction equilibrium or divergent trajectory may occur and the outcome depends on initial conditions. Lastly, when both conditions above are false (i.e. $\tilde{R} > R_1^* > R_1^c$), then the system with the producer in isolation has a single non-zero equilibrium that is stable. In this case, the producer can either receive a net benefit or net cost from resource production, but behaves as a net consumer at equilibrium.

Now, consider the two species in competition. In the case where the producer is a net consumer (no increase to infinity), we can simply compare R_1^* and R_2^*: the species with the smaller R^* excludes the other (the R^* rule; [5, 6, 7]). If parameters are such that the two species have the same R^* (which happens when $\alpha = \gamma$, and when the production function is zero at R_2^*), then they are competitively neutral (though not physiologically equivalent) and coexist neutrally. Finally, when the producer diverges, coexistence may occur with the two species diverging to infinity.

References

1. Hardin G. The competitive exclusion principle; 1960.
2. Levin SA. Community equilibria and stability, and an extension of the competitive exclusion principle. American Naturalist. 1970;104(939):413–423. doi:10.2307/2678832.

3. Meszéna G, Gyllenberg M, Pásztor L, Metz JAJ. Competitive exclusion and limiting similarity: a unified theory. Theoretical Population Biology. 2006;69(1):68–87. doi:10.1016/j.tpb.2005.07.001.

4. Koffel T, Daufresne T, Klausmeier CA. From competition to facilitation and mutualism: a general theory of the niche. Ecological Monographs. 2021;0(0):1–31. doi:10.1002/ecm.1458.

5. Hsu SB, Hubbell S, Waltman P. A mathematical theory for single-nutrient competition in continuous cultures of micro-organisms. SIAM Journal on Applied Mathematics. 1977;32(2):366–383. doi:10.1137/0132030.

6. Tilman D. Resource Competition and Community Structure. Princeton, NJ: Princeton University Press; 1982.

7. Chase JM, Leibold MA. Ecological Niches: Linking Classical and Contemporary Approaches. Chicago: University of Chicago Press; 2003.
