# Supplementary material for: How public can public goods be? Environmental context shapes the evolutionary ecology of partially private goods
Source: PLoS Comput Biol. 2022 Nov 1;18(11):e1010666. doi: 10.1371/journal.pcbi.1010666 (PMC9651594; doi:10.1371/journal.pcbi.1010666)
Supplement: S2 Appendix — (PDF) [file pcbi.1010666.s010.pdf]

S2 Appendix: Analytical results in the limit of one resource in excess

Brian A. Lerch, Derek A. Smith, Thomas Koffel, Sarah C. Bagby, Karen C. Abbott

In this Appendix, we provide analytical results from the colimitation model in the cases where a single resource limits population growth.

We begin by specifying a numerical constraint on our parameters that is needed to obtain analytical results and for interpretation. We assume decreasing production will not outpace increasing uptake; mathematically, this requires that

$$\frac{ad}{(d+N)^2} > \frac{\alpha \ell b}{(\ell+N)^2} \tag{S2.1}$$

for any positive N, which can be shown to lead to the simple condition of

$$\frac{a}{\alpha b} > \frac{d}{\ell} > \frac{\alpha b}{a}. \tag{S2.2}$$

Notably, Eq (S2.2) can only be true if $a > \alpha b$: for increased fixed nitrogen availability to increase fixed nitrogen acquisition, acquisition under unlimited nitrogen (equal to a, the maximal uptake rate) has to be greater than acquisition under complete absence of nitrogen (equal to αb, the maximal fixation rate). Then, Eq (S2.2) states that total fixed nitrogen acquisition always increases with N when the ratio of the half-inhibition and half-saturation constants is between the ratios of the maximal production and maximal uptake rates. We ensured that only parameter sets that followed this constraint were used in the sensitivity analysis.

We now turn toward understanding the case of single-resource limitation. The ZNGI of a strain is obtained by setting its growth rate equal to zero:

$$0 = \mathscr{G}(S,N) - \gamma f_N^i(N) - \beta f_S^i(S) - \delta. \tag{S2.3}$$

We rewrite the ZNGI equation as

$$0 = \frac{r\eta_i(N)}{\eta_i(N) + r(\kappa + v/S)} - \gamma f_N^i(N) - \beta f_S^i(S) - \delta \tag{S2.4}$$

to reduce the number of non-free parameters, and better showcase parameter dimensionality ($\kappa = (1+k_S)/c$ has no units, $v = k_S p/c$ has the units of S). Notably, this ZNGI has two asymptotes, one as $S \to \infty$ where cell growth is limited only by N, and one as $N \to \infty$ where cell growth is limited by S.

N-limited regime

The first asymptote is horizontal in $S-N$ space, obtained when $S \to \infty$, at a value we call N^* (the minimal amount of nitrogen a strain can grow on when S is not limiting). N^* satisfies the implicit equation

$$0 = \frac{r\eta_i(N^*)}{\eta_i(N^*) + r\kappa} - \gamma f_N^i(N^*) - \delta. \tag{S2.5}$$

Notice that all terms dealing with siderophore production drop out of Eq (S2.5) and thus N^* does not depend on whether or not a strain produces siderophores. Unfortunately, this is not explicitly solvable, but we can study the effect of fixation on N^* by looking at the sign of the derivative dN^*/db_i. This computation starts with rewriting the implicit equation as

$$0 = \mathscr{G}(\eta_i(N^*)) - \gamma f_N^i(N^*) - \delta, \tag{S2.6}$$

where $\mathscr{G}(\eta_i(N^*))$ denotes $\mathscr{G}(N^*, \infty) = r\eta_i(N^*)/(\eta_i(N^*) + r\kappa)$, a type II functional response in η_i. Then, differentiating both sides of this equality gives

$$\begin{aligned}
0 &= \frac{\partial \mathscr{G}}{\partial \eta_i}\left(\frac{\partial \eta_i}{\partial N}dN + \frac{\partial \eta_i}{\partial b_i}db_i\right) - \gamma\left(\frac{\partial f_N^i}{\partial N}dN + \frac{\partial f_N^i}{\partial b_i}db_i\right) &\text{(S2.7)}\\
&= \frac{\partial \mathscr{G}}{\partial \eta_i}\left[\left(\alpha\frac{\partial f_N^i}{\partial N} + \frac{\partial U_N}{\partial N}\right)dN + \alpha\frac{\partial f_N^i}{\partial b_i}db_i\right] - \gamma\left(\frac{\partial f_N^i}{\partial N}dN + \frac{\partial f_N^i}{\partial b_i}db_i\right) &\text{(S2.8)}\\
&= \left[\frac{\partial \mathscr{G}}{\partial \eta_i}\left(\alpha\frac{\partial f_N^i}{\partial N} + \frac{\partial U_N}{\partial N}\right) - \gamma\frac{\partial f_N^i}{\partial N}\right]dN + \left(\alpha\frac{\partial \mathscr{G}}{\partial \eta_i}\frac{\partial f_N^i}{\partial b_i} - \gamma\frac{\partial f_N^i}{\partial b_i}\right)db_i &\text{(S2.9)}
\end{aligned}$$

from which we can single out the derivative $\partial N^*/\partial b_i$, after some term reordering and factorization, to obtain

$$\frac{\partial N^*}{\partial b_i} = -\frac{\left(\alpha\frac{\partial \mathscr{G}}{\partial \eta_i} - \gamma\right)\frac{\partial f_N^i}{\partial b_i}}{\left(\alpha\frac{\partial \mathscr{G}}{\partial \eta_i} - \gamma\right)\frac{\partial f_N^i}{\partial N} + \frac{\partial \mathscr{G}}{\partial \eta_i}\frac{\partial U_N}{\partial N}}. \tag{S2.10}$$

Though this appears complicated, there is a very simple interpretation of this derivative. Note that $\frac{\partial \mathscr{G}}{\partial \eta_i}$ and $\frac{\partial U_N}{\partial N}$ are both non-negative (growth and uptake increase with nitrogen availability) and $\frac{\partial f_N^i}{\partial N}$ is non-positive (available N inhibits fixation). In addition, the denominator of the above expression (which is the change in overall per capita growth rate with nitrogen availability) is always positive, as increasing N always has a positive net effect on growth (similar general assumptions were made for the single resource model, and always met here for our specific choice of production and uptake functions). Then, Eq (S2.10) is negative (notice the minus sign in front of the fraction) when

$$\left.\frac{\partial \mathscr{G}}{\partial \eta_i}\right|_{\substack{S\to\infty\\ N=N^*}} = \mathscr{S}(\infty, N^*) > \frac{\gamma}{\alpha}, \tag{S2.11}$$

where we have defined $\mathscr{S}(S', N') = \left.\frac{\partial \mathscr{G}}{\partial \eta_i}\right|_{\substack{S=S'\\ N=N'}}$ as the sensitivity of growth to nitrogen acquisition (see main text; Eq (14)). Very similarly to the interpretation below, this tells us that increased fixation is adaptive (lowers N^*) when the realized benefits (the direct benefits α weighted by the

sensitivity of growth to increased N availability, $\mathscr{S}$; note that there are no direct benefits of fixation when growth has already saturated at its type II plateau at N^*) are larger than the direct costs, γ. Notice that the quantity $\mathscr{S}$ is complicated, depends on multiple parameters, and must be evaluated at N^*.

A particularly noteworthy case is obtained by setting $b_i = 0$, so that $f_N^i = 0$, i.e., by looking at a mutant that does not fix N (LOFN or LOFB). Then, the condition on $\partial N^*/\partial b_i$ tells us if it is advantageous or not for such a strain to turn on fixation when solely N is limited. In that case, N^* is given by solving

$$0 = \frac{rU_N(N^*)}{U_N(N^*) + r\kappa} - \delta, \tag{S2.12}$$

i.e.,

$$N^* = U_N{}^{-1}\left(\frac{r\delta}{r-\delta}\kappa\right) \tag{S2.13}$$

Then, we have

$$\mathscr{S}(\infty, N^*) = \frac{1}{\kappa}\frac{r-\delta}{r}. \tag{S2.14}$$

The condition for a small amount of fixation being beneficial compared to not fixing is now straightforward:

$$\frac{1}{\kappa}\frac{r-\delta}{r} > \frac{\gamma}{\alpha}. \tag{S2.15}$$

We see here how the saturating nature of the type II functional response of $\mathscr{G}$ to nitrogen availability η_i (here $\eta_i = U_N$ as fixation is zero) influences how much direct benefits can be derived from fixation. When taking the limit $r \to \infty$, this type II functional response becomes a non-saturating type I and the fraction on the LHS simplifies to 1. This highlights how growth saturation through finite r decreases the potential for growth, and thus the direct benefits of fixation, as the difference $r - \delta$ gets smaller.

S-limited regime

The second asymptote, called S^*, is obtained when $N \to \infty$, satisfying the implicit equation

$$0 = \mathscr{G}(\infty, S^*) - \gamma f_N^i(\infty) - \beta f_S^i(S^*) - \delta = \frac{ra}{a + r(\kappa + v/S^*)} - \beta\frac{q_i}{m + S^*} - \delta \tag{S2.16}$$

because $f_N^i(N) \to 0$ and $\eta_i(N) \to a$ when $N \to \infty$. This can be solved, but the result is too complex to be useful, so we mostly use the implicit equation. However, let us first show that production of siderophores can only raise a strain's S^* (and thus make it a worse competitor when siderophores are limiting). This is done by differentiating both sides of Eq (S2.16), leading to

$$0 = \frac{\partial S^*}{\partial q_i}\frac{\partial \mathscr{G}}{\partial S} - \beta\left(\frac{\partial f_S^i}{\partial q_i} + \frac{\partial f_S^i}{\partial S}\frac{\partial S^*}{\partial q_i}\right) \tag{S2.17}$$

which gives

$$\frac{\partial S^*}{\partial q_i} = \frac{\beta \frac{\partial f_S^i}{\partial q_i}}{\frac{\partial \mathcal{G}}{\partial S} - \beta \frac{\partial f_S^i}{\partial S}}. \tag{S2.18}$$

Despite its notational complexity, this result is simple to interpret if we remember that $\partial f_S^i / \partial q_i > 0$ and $\partial \mathcal{G} / \partial S - \beta \partial f_S^i / \partial S > 0$ (S availability has a net positive effect on growth), which means that we always have

$$\frac{\partial S^*}{\partial q_i} > 0 \tag{S2.19}$$

which completes the proof that siderophore production is always detrimental for a species' competitive ability when N is non-limiting. In fact, this result is true for any value of S of the ZNGI, not only S^*, i.e., when N takes any value. Thus, increasing q_i moves all the points on the ZNGI to the right for fixed N.

Importantly, as can be seen in the implicit equation, S^* is completely independent from the parameters that control fixation. Let us look at the next order of the asymptotic behavior of the ZNGI around S^* to see an effect of fixation.

A differentiation-based procedure similar to the one above used for N^* can be applied to study how fixation b_i affects the relationship between S and N in the neighborhood of S^*:

$$\frac{\partial S}{\partial N} = -\frac{\left(\alpha \frac{\partial \mathcal{G}}{\partial \eta_i} - \gamma\right) \frac{\partial f_N^i}{\partial N} + \frac{\partial \mathcal{G}}{\partial \eta_i} \frac{\partial U_N}{\partial N}}{\frac{\partial \mathcal{G}}{\partial S} - \beta \frac{\partial f_S^i}{\partial S}}. \tag{S2.20}$$

The steeper this slope, the more competitive the strain is in the vicinity of $(S^*, N \to \infty)$. More precisely, because only f_N^i depends on b_i in the equation above, the net competitive advantage of fixation is given by:

$$\left.\frac{\partial S}{\partial N}\right|_{b_i \neq 0} - \left.\frac{\partial S}{\partial N}\right|_{b_i = 0} = -\frac{\left(\alpha \frac{\partial \mathcal{G}}{\partial \eta_i} - \gamma\right) \left.\frac{\partial f_N^i}{\partial N}\right|_{b_i \neq 0}}{\frac{\partial \mathcal{G}}{\partial S} - \beta \frac{\partial f_S^i}{\partial S}}. \tag{S2.21}$$

Now, as $\left.\frac{\partial f_N^i}{\partial N}\right|_{b_i \neq 0} < 0$ and the denominator of the fraction above is positive (increasing S always has a positive net effect on growth), we see that N-fixation b_i is advantageous (makes the slope near S^* steeper) when

$$\left.\frac{\partial \mathcal{G}}{\partial \eta_i}\right|_{\substack{S=S^* \\ N \to \infty}} = \mathcal{S}(S^*, \infty) > \frac{\gamma}{\alpha}. \tag{S2.22}$$

This is the same criterion as above, but notably, $\mathcal{S}$ has to be evaluated at $(S^*, N \to \infty)$, where the benefits of increasing fixation are likely to be considerably smaller than the previous case where $N^* \to \infty$. However, if costs are sufficiently small, fixation may be beneficial in the vicinity of S^*. Formally, we expect

$$\left.\frac{\partial \mathcal{G}}{\partial \eta_i}\right|_{\substack{S \to \infty \\ N = N^*}} = \mathcal{S}(\infty, N^*) \gg \mathcal{S}(S^*, \infty) \left.\frac{\partial \mathcal{G}}{\partial \eta_i}\right|_{\substack{S=S^* \\ N \to \infty}} \tag{S2.23}$$

so that the second condition we found for a benefit to fixation when S is limiting (inequality (S2.22)) is harder to realize than the one we found in the last subsection for a benefit to fixation when N is limiting (inequality (S2.11)).
