# Supplementary material for: How public can public goods be? Environmental context shapes the evolutionary ecology of partially private goods
Source: PLoS Comput Biol. 2022 Nov 1;18(11):e1010666. doi: 10.1371/journal.pcbi.1010666 (PMC9651594; doi:10.1371/journal.pcbi.1010666)
Supplement: S3 Appendix — (PDF) [file pcbi.1010666.s011.pdf]

S3 Appendix: The relative ordering of impact vectors

Brian A. Lerch, Derek A. Smith, Thomas Koffel, Sarah C. Bagby, Karen C. Abbott

We now turn to assessing the relative ordering of impact vectors from a fixed but arbitrary resource concentration (S', N'). When (S', N') are coexistence equilibria, this analysis clarifies whether two strains coexist stably versus be subject to a priority effect. Arguments about the realitve order of impact vectors hold for any values of S and N and do not rely upon these being equilibrium concentrations. Throughout, for simplicity, we will treat (S', N') as the origin, but of course $S, N > 0$ is required for biological realism (i.e., we translate the $S-N$ plane such that (S', N') is the origin). Treating (S', N') as the origin allows us to define the following. The first quadrant in the $S-N$ plane are resource concentrations such that $S > S'$ and $N > N'$. The second quadrant in the $S-N$ plane are resource concentrations such that $S < S'$ and $N > N'$. The third quadrant in the $S-N$ plane are resource concentrations such that $S < S'$ and $N < N'$. And the fourth quadrant in the $S-N$ plane are resource concentrations such that $S > S'$ and $N < N'$.

We begin with LOFB. The impact vector for LOFB has slope

$$m_{\text{LOFB}} = \frac{U_N(N')}{U_S(S')}. \tag{S3.1}$$

The fact that it is always positive means that the impact vector points down and to the left in the $S-N$ plane. This matches biological intuition because LOFB can only be a net consumer, since it does not produce either resource.

The impact vector for LOFN has slope

$$m_{\text{LOFN}} = \frac{U_N(N')}{U_S(S') - f_S^i(S')}. \tag{S3.2}$$

Notice that m_{LOFB} and m_{LOFN} have the same numerator. Thus, LOFN has a larger slope than LOFB so long as $m_{\text{LOFN}} > 0$. It is capable of having a negative slope in the second quadrant (i.e., pointing right from S concentrations below S'), but its angle from the positive S-axis can never exceed π radians, since this would imply that it is a net producer of nitrogen.

The impact vector for LOFS has slope

$$m_{\text{LOFS}} = \frac{U_N(N') - f_N(N')}{U_S(S')}. \tag{S3.3}$$

Notice that m_{LOFB} and m_{LOFS} have the same denominator. Thus, LOFS has a smaller slope than LOFB so long as $m_{\text{LOFS}} > 0$. It is capable of having a negative slope in the fourth quadrant (i.e., pointing up from N concentrations below N'), but its angle from the positive S-axis can never exceed $-\pi/2$ radians, since this would imply that it is a net producer of siderophores.

In sum, this implies (ignoring Full) that moving from the negative S-axis clockwise, the first impact vector will always belong to LOFN, the second LOFB, and the third LOFS. Or, equivalently, moving from the negative N-axis counterclockwise will pass through the impact vectors in the order of LOFS, LOFB, then LOFN.

Now, we will consider the relative position of the fully-functional strain's impact vector. Of course, the slope is

$$m_{\text{Full}} = \frac{U_N(N') - f_N(N')}{U_S(S') - f_S(S')}. \tag{S3.4}$$

The challenge is that this strain can either be a net producer or a net consumer of either resource. Thus, its impact vector is capable of pointing in any direction in the resource plane. One can, however, assess four special cases based on whether the full strain is a net producer or consumer of either resource. By considering just the effect of changing f_N, one can compare m_{Full} to m_{LOFN} (left two panels of S7 Fig a). Likewise, by considering jsut the effect of f_S, m_{Full} can be compared to m_{LOFS} (right two panels of S7 Fig a). This allows for a complete picture of how the impact vector of the fully-functional strain compares to LOFN and LOFS (S7 Fig b).

Now, we will use the relative positions of the impact vectors to determine whether the coexistence equilibria between the various strains are stable or unstable (a priority effect). We ignore the competitive pairs where no coexistence equilibrium exists (LOFN vs LOFB and Full vs LOFS). We also omit competitive pairs that include the fully-functional strain, since the flexibility of its impact vector precludes general statements about coexistence. The remaining competitive pairs are considered below.

LOFS vs LOFN There are two possible coexistence equilibria for LOFS and LOFN. First, the case in Fig 4civ implies that fixation is a net cost when nitrogen is limiting. Thus, LOFN can exclude LOFS in the N-limited case (LOFS always excludes LOFN in the S-limited case). Because the impact vector for LOFS is always "below" the impact vector for LOFN, then this implies that coexistence between these two strains cannot be stable and we get priority effects instead. Second, the case in S5 Fig implies that the benefit of fixation depends on the environment. Here, LOFS always wins in the limit of S^*, but LOFN wins moving away from S^* (S5 Fig b). Due to arguments about the ordering of impact vectors presented above, this implies that this "upper" equilibrium will always be unstable. Similarly, as we move towards N-limitation, LOFS begins to again outcompete LOFN (S5 Fig c). Again, arguments about the ordering of impact vectors implies that this will be a stable coexistence equilibrium. Notice that it is also possible for either one or both strains to be net producers at equilibrium, which, if their production becomes too strong, can lead each producer to overcome self-limitation (which happens when a strain's impact vector gets "below" its ZNGI), turning that strain's monoculture equilibrium into single species' unbounded growth. When this happens, the priority effect between the two stable monocultures is replaced by either a priority effect between one stable monoculture and one single-species unbounded growth trajectory, or between two single-species unbounded growth trajectories; see Fig S2D,E and S3 in [1]'s Appendix S6.

LOFS vs LOFB If there is a coexistence region for LOFS and LOFB, then this implies that fixation is a net benefit when nitrogen is limiting, but a net cost when siderophores are limiting.

Thus, LOFB can exclude LOFS in the S-limited case and LOFS can exclude LOFB in the N-limited case. Because the impact vector for LOFS is always “below” the impact vector for LOFB, then this implies that coexistence between these two strains is stable (unless LOFS produces enough nitrogen to overcome self-limitation, in which case coexistence can be destabilized; see Fig S2B in [1]’s Appendix S6).

References

1. Koffel T, Daufresne T, Klausmeier CA. From competition to facilitation and mutualism: a general theory of the niche. Ecological Monographs. 2021;0(0):1–31. doi:10.1002/ecm.1458.
