# Supplementary material for: How public can public goods be? Environmental context shapes the evolutionary ecology of partially private goods
Source: PLoS Comput Biol. 2022 Nov 1;18(11):e1010666. doi: 10.1371/journal.pcbi.1010666 (PMC9651594; doi:10.1371/journal.pcbi.1010666)
Supplement: S4 Appendix — (PDF) [file pcbi.1010666.s012.pdf]

S4 Appendix: Adaptive dynamics in the colimitation model

Brian A. Lerch, Derek A. Smith, Thomas Koffel, Sarah C. Bagby, Karen C. Abbott

Under the assumptions of adaptive dynamics (Box 2), analytical results can be obtained for the evolution of some traits in our model.

Siderophore production rate

We begin by considering the evolution of the siderophore production rate. We assume that the cell population is nearly monomorphic with siderophore production rate q_R and consider an invader with siderophore production rate q_I. Define S_R^* and N_R^* to be the siderophore and nitrogen concentrations, respectively, at the resident's equilibrium. Then, the invasion growth rate (Box 2) is

$$G(q_I, q_R) = \mathscr{G}_I(S_R^*, N_R^*) - \gamma f_N^I(N_R^*) - \beta f_S^I(S_R^*) - \delta. \tag{S4.1}$$

Since only f_S^I depends upon q_i, computing the fitness gradient simply gives

$$\left.\frac{\partial G}{\partial q_I}\right|_{q_I=q_R} = -\beta \left.\frac{\partial f_S^I}{\partial q_I}\right|_{q_I=q_R} = -\frac{\beta m}{m + S_R^*}. \tag{S4.2}$$

Clearly, Eq (S4.2) is negative. Thus, siderophore production is always disfavored and lower siderophore production rates will always invade under the assumptions of adaptive dynamics.

Privatization

Though we assumed that cell strains do not differ in their privatization in the main text, one can readily incorporate interstrain variation in privatization into the model. We assume that cell strains are equivalent except that the degree of privatization of fixation is α_i for strain i. Doing so under the assumptions of adaptive dynamics allows for an assessment of how privatization will evolve. Again, the invasion growth rate is given by Eq (S4.1), but note that now sub- and superscript I denotes an invading strain with the resident's value of q and with privatization α_I. Note that privatization only alters the colimitation growth term $\mathscr{G}_i$. Thus, the fitness gradient is

$$\left.\frac{\partial G}{\partial \alpha_I}\right|_{\alpha_I=\alpha_R} = \left.\frac{\partial \mathscr{G}_I}{\partial \alpha_I}\right|_{\alpha_I=\alpha_R} = \frac{cr^2(k_S + U_S^I(S_R^*)) f_N^I(N_R^*) U_S^I(S_R^*)}{k_S r U_S^I(S_R^*)(r + c(U_N^I(N_R^* + \alpha_R f_N^I(N_R^*))))^2}. \tag{S4.3}$$

Though complex, clearly every term of Eq (S4.3) is positive and thus Eq (S4.3) is positive. This means that if privatization were an evolvable trait under the assumptions of adaptive dynamics, then privatization would evolve to be greater.
